# Blood and Urine Transcriptomes Reveal Complementary Host Responses and a Creatinine-Associated Urinary Transcriptional Axis in Sepsis

**DOI:** 10.3390/diagnostics16203410

**Published:** 2026-10-20

**Authors:** Vyacheslav Beloussov, Vitaliy Strochkov, Nurlan Sandybayev, Alyona Lavrinenko, Talgat Aitpenov, Maxim Solomadin

**Affiliations:** 1 Kazakhstan-Japan Innovation Centre, Kazakh National Agrarian Research University, Almaty 050010, Kazakhstan; 2 Scientific Research Laboratory, Karaganda Medical University, Karaganda 100012, Kazakhstan; 3 Multidisciplinary Hospital Named After Professor H.J. Makazhanov, Karaganda 100008, Kazakhstan; 4 International Center for Vaccinology, Kazakh National Agrarian Research University, Almaty 050010, Kazakhstan

**Keywords:** sepsis, host response, blood transcriptomics, urinary transcriptomics, targeted RNA sequencing, differential gene expression, serum creatinine, creatinine-associated transcriptional axis, multi-biofluid transcriptomics

## Abstract

**Background/Objectives**: Sepsis is characterized by systemic immune dysregulation and organ dysfunction, but transcriptomic profiles from different biofluids may provide partially overlapping and distinct molecular readouts of the host response. We characterized transcriptomic profiles of whole blood and urinary cellular fractions in sepsis, with particular emphasis on a urinary transcriptional gradient associated with continuous serum creatinine and on whether this association was mirrored in matched blood. **Methods**: Targeted RNA sequencing was performed on whole blood from 35 patients with sepsis and 10 healthy controls and on urinary cellular fractions from 21 patients with sepsis and 7 controls using the Ion AmpliSeq Transcriptome Human Gene Expression workflow. Differential expression was assessed with DESeq2, with sequencing-adjusted sensitivity models where appropriate. Sensitivity analyses addressed the highest creatinine value, sex and age, and matched blood–urine creatinine-associated effects. **Results**: Whole-blood analysis identified 2659 differentially expressed genes (DEGs), including a robust core of 1796 genes characterized by neutrophil–myeloid activation and adaptive-immune suppression. The exploratory urine case–control comparison identified 1552 DEGs but was substantially confounded by sequencing chip. Continuous serum creatinine analysis identified 4064 chip-adjusted urinary DEGs, including a robust core of 3774 genes enriched for inflammatory, interferon, hypoxic, autophagic, lysosomal, vesicular, metabolic, and mitochondrial programs. Exclusion of the highest creatinine sample retained 3192/4064 strict DEGs with the same direction and 2926/3774 robust-core genes. In the same 21 patients, adjusted creatinine-associated effects were weakly concordant between blood and urine at the gene level (Pearson r = 0.192; Spearman ρ = 0.199), with five overlapping strict DEGs and no robust-core overlap. Hallmark concordance was greater but moderate (Pearson r = 0.293; Spearman ρ = 0.464), with 14 shared robust pathways. **Conclusions**: Transcriptomic profiles of whole blood and urinary cellular fractions showed distinct but partially overlapping host-response patterns in sepsis. The serum creatinine-associated urinary transcriptional axis was robust across sensitivity analyses and was only weakly mirrored at the gene level in matched blood, despite partial pathway-level convergence. These findings should be interpreted as serum creatinine-associated transcriptional patterns rather than as evidence of kidney-specific injury or validated diagnostic or prognostic signatures. Independent longitudinal cohorts and orthogonal molecular validation are required.

## 1. Introduction

Sepsis is life-threatening organ dysfunction caused by a dysregulated host response to infection and remains a major cause of mortality worldwide [1,2,3,4]. Tens of millions of cases of sepsis are reported annually, accounting for a significant proportion of global mortality [1,3]. Despite advances in intensive care and antimicrobial therapy, sepsis remains a significant problem associated with high mortality, prolonged hospitalization, and persistent physical and cognitive impairments in surviving patients [2,5,6].

Available national data identify respiratory, intra-abdominal, and urinary tract infections as major sources of bacteremia and indicate a clinically relevant prevalence of antimicrobial-resistant pathogens, including *Staphylococcus aureus*, methicillin-resistant *S. aureus*, and extended-spectrum β-lactamase-producing Enterobacterales [7]. These findings underscore the need for locally generated molecular data.

The pathophysiology of sepsis involves interacting immune, endothelial, coagulation, metabolic, and organ dysfunction pathways, and the host response is highly heterogeneous [8]. Patients may exhibit hyperinflammatory, immunosuppressive, endothelial, coagulopathic, or mixed biological phenotypes [9,10]. This heterogeneity may partly explain the inconsistent efficacy of immunomodulatory therapies [11,12,13]. No rapid test currently combines reliable identification of infection-related critical illness, characterization of the host response, and prediction of organ dysfunction [14,15,16,17,18]. Accordingly, precision medicine strategies increasingly aim to define biologically distinct patient subgroups for improved risk stratification and targeted treatment [9,10,12,19,20,21,22,23].

Transcriptomic profiling is a leading systems biology approach for studying the heterogeneity of sepsis [9,10,24]. Blood gene expression signatures can identify endotypes associated with disease severity, immune dysfunction, treatment response, and mortality [9,10,18,23,25,26,27]. Reported endotypes include immunosuppressive, innate- or adaptive-immune, interferon, coagulopathic, and endothelial programs [9,10,23,26,28], some of which have demonstrated potential value for predicting corticosteroid response, immunomodulatory outcomes, and short-term mortality [25,27,29,30].

However, most transcriptomic studies of sepsis have focused on peripheral blood [26,27,28,29,30]. Although blood reflects systemic immune activation, it may not fully capture molecular processes represented in other biological compartments [31,32,33]. This limitation is clinically relevant because acute kidney injury (AKI) is common in sepsis and strongly associated with adverse outcomes [32,33]. It occurs in 40–50% of patients with sepsis and is associated with a substantially increased risk of adverse outcomes. Early sepsis-associated kidney injury may precede marked changes in serum creatinine and urine output, limiting the sensitivity of conventional clinical indicators [31]. Serum creatinine nevertheless remains a routinely available clinical measure of renal function, although it is not specific for kidney injury and may also be influenced by systemic illness and other patient-related factors.

Urine is an accessible, non-invasive source of molecular information in critically ill patients [34,35]. Cellular and extracellular RNA in urine may reflect systemic inflammation, glomerular and tubular injury, and kidney-related pathological mechanisms [32,35]. Previous studies suggest that urine transcriptomics can distinguish sepsis from sterile inflammation and reveal immune, metabolic, oxidative stress, and tissue remodeling pathways [36]. Single-cell analyses further indicate that shed urinary cells can reflect states of kidney injury and repair [32]. However, bulk urinary transcriptomic profiles are also influenced by the abundance and composition of shed cells and therefore cannot, without cellular-resolution data, be attributed exclusively to kidney-specific molecular processes.

Integrating blood and urine transcriptomes in sepsis may therefore help characterize shared and biofluid-specific patterns of the host response [37]. Blood primarily reflects circulating immune activity, whereas urinary transcriptomic profiles may additionally contain signals derived from urinary tract cells, local immune responses, and filtered molecular material, while also being strongly influenced by differences in cellular composition [35]. Nevertheless, differences in cohort composition, sample processing, sequencing platforms, and analytical pipelines limit reproducibility [20], and many proposed transcriptomic classifiers still lack standardization and independent validation [9].

In this study, we compared targeted RNA-sequencing profiles from blood and urine in patients with sepsis. We aimed to identify shared and biofluid-specific molecular programs and to characterize a urinary transcriptomic axis associated with serum creatinine. Our findings identify partially overlapping but distinct blood and urinary transcriptomic patterns in sepsis and a robust urinary transcriptional gradient associated with serum creatinine.

## 2. Materials and Methods

### 2.1. Study Design

This prospective observational study compared transcriptomic profiles from patients with sepsis and healthy controls and included nested exploratory clinical–transcriptomic analyses within the sepsis cohort. No randomization, experimental intervention, or alteration of standard clinical care was undertaken.

Targeted RNA sequencing was used to profile whole blood and the cellular fraction of urine. Blood was evaluated as a source of systemic immune response signals, while urine was investigated as a noninvasive source of epithelial, immune, metabolic, and stress-related molecular signals.

The primary analyses compared sepsis and control samples separately in blood and urine and assessed paired blood–urine profiles from patients with sepsis. An additional exploratory analysis examined associations between gene expression in the urinary cellular fraction and serum creatinine as a continuous clinical measure related to renal function and subsequently compared creatinine-associated effects between matched blood and urine samples.

Serum creatinine was analyzed both categorically and continuously. For the categorical analysis, patients were stratified using a study-defined threshold of 110 μmol/L, selected with reference to the 110 μmol/L boundary of the renal Sequential Organ Failure Assessment (SOFA) subscore (>110 versus ≤110 μmol/L). The continuous model was used to assess whether urinary gene expression varied along the serum creatinine gradient rather than only between the predefined groups. Overall, the study aimed to identify shared and biofluid-specific molecular programs and to characterize a urinary transcriptomic axis associated with serum creatinine in sepsis.

### 2.2. Study Population and Clinical Criteria

Patients were prospectively and consecutively recruited between 18 November 2024 and 17 September 2025 at three hospitals in Karaganda, Kazakhstan: Karaganda Multidisciplinary Hospital No. 1, Professor Kh. Zh. Makazhanov Multidisciplinary Hospital, and Karaganda Regional Clinical Hospital. Sequential study identifiers were assigned at enrollment.

A total of 100 adults aged ≥18 years with suspected or clinically diagnosed sepsis were screened. Recruitment and clinical classification were conducted in accordance with the national Clinical Protocol for the Diagnosis and Treatment of “Sepsis,” approved by the Joint Commission on the Quality of Medical Services of the Ministry of Health of the Republic of Kazakhstan on 1 April 2020 (Protocol No. 89). Sepsis was defined as a suspected or documented infection accompanied by acute organ dysfunction, indicated by an increase in the SOFA score of ≥2 points from baseline. The diagnosis was established by the treating clinical team using routine clinical, laboratory, microbiological, and diagnostic imaging findings.

Biological specimens were collected from 65 patients, 35 of whom were included in the final RNA-seq cohort. Patients were excluded if they were younger than 18 years of age, if the final diagnosis of sepsis was not confirmed, if biological material was unavailable or insufficient, if samples failed to meet laboratory quality requirements, or if clinical annotation was inadequate for transcriptomic analysis.

The control group consisted of 10 healthy adult volunteers recruited at Karaganda Medical University in Karaganda, Kazakhstan. Blood and urine samples were collected from all controls on 2 May 2025. The controls showed no evidence of acute or recent infection, systemic or chronic inflammatory disease, chronic kidney disease, or current immunomodulatory therapy.

Whole-blood RNA sequencing was performed on 35 patients with sepsis and 10 controls. The urinary cellular fraction was profiled in 21 patients with sepsis and 7 controls whose samples met the RNA quantity and quality requirements. Paired blood–urine RNA-seq profiles were therefore available for 21 patients with sepsis and 7 controls.

### 2.3. Ethics Approval and Informed Consent

The study was conducted in accordance with the Declaration of Helsinki and the national laws of the Republic of Kazakhstan governing biomedical research involving human participants. The study protocol was approved by the Local Bioethics Committee of Karaganda Medical University (Protocol No. 14, 17 November 2023).

Prior to enrollment, all participants or their legally authorized representatives were informed about the study objectives, the collection of biological samples, the planned laboratory and molecular analyses, and the procedures for data processing, storage, and protection. Written informed consent was obtained prior to the collection of biological samples.

Biological samples and clinical and laboratory data were de-identified prior to laboratory and bioinformatics analyses. Access to individual-level source data was restricted to authorized members of the research team.

### 2.4. Sample Collection and RNA Extraction

Blood and urine samples from patients with sepsis were collected within 1.5 h after the preliminary clinical diagnosis, according to the study sample-collection protocol developed with reference to the national Clinical Protocol for Diagnosis and Treatment “Sepsis” (Protocol No. 89, approved 1 April 2020). Sample collection was performed by trained medical personnel in accordance with institutional biosafety procedures.

Three milliliters of venous whole blood were collected into Tempus™ Blood RNA Tubes containing 6 mL of RNA-stabilizing reagent (cat. no. 4342792; Applied Biosystems™, Thermo Fisher Scientific, Vienna, Austria). Immediately after collection, the tubes were vigorously mixed for 10 s to ensure complete contact between the blood and the stabilizing reagent. Tempus™ tubes were temporarily stored at 2–8 °C and subsequently frozen at −70 °C for long-term storage. Collecting 3 mL of whole blood and storing it for up to 7 days at 4 °C before long-term freezing is consistent with the manufacturer’s recommendations.

Up to 50 mL of freshly collected urine was placed in sterile single-use containers in accordance with standard clinical collection procedures. The method of urine collection–spontaneous voiding or collection via an indwelling urinary catheter–was not consistently recorded and was therefore not used as an analytical variable. Urine sediment cellular composition and routine urine microscopy data, including hematuria and pyuria, were not available for the RNA-seq cohort. Urine samples were stored at 2–8 °C and processed within 2 h of collection.

The urine was transferred to RNase- and DNase-free 50 mL conical tubes (cat. no. AM12502; Invitrogen™/Ambion™, Thermo Fisher Scientific, Austin, TX, USA) and centrifuged at 1500× *g* for 30 min at 4 °C using a Sigma 3-30KS refrigerated centrifuge (Sigma Laborzentrifugen GmbH, Osterode am Harz, Germany). After completely removing the supernatant, the urinary cell pellet was resuspended in 1 mL of RNAlater™ Stabilization Solution (cat. no. AM7020; Invitrogen™/Ambion™, Thermo Fisher Scientific, Vilnius, Lithuania), incubated overnight at 4 °C, and stored at −70 °C until RNA extraction.

Samples were coded according to biofluid type and study group: BP, blood from sepsis patients; UP, urine from sepsis patients; BC, control blood; and UC, control urine. Initial sample processing and storage were performed at the Research Laboratory of Karaganda Medical University in Karaganda. Frozen, stabilized samples were transported on dry ice to the Kazakhstan-Japan Innovation Center at the Kazakh National Agrarian Research University in Almaty within 24 h, in accordance with applicable biosafety requirements for the transport of biological specimens.

Total RNA was extracted from Tempus™-stabilized whole blood using the Tempus™ Spin RNA Isolation Kit (cat. no. 4380204; Applied Biosystems™/Invitrogen™, Thermo Fisher Scientific, Inchinnan, UK). RNA from urinary cell pellets was extracted using the MagMAX™ mirVana™ Total RNA Isolation Kit (cat. no. A27828; Applied Biosystems™, Thermo Fisher Scientific, Vilnius, Lithuania), according to the manufacturers’ protocols.

RNA concentration was measured by fluorometry using the Qubit™ RNA HS Assay Kit (cat. no. Q32852; Invitrogen™, Thermo Fisher Scientific, Eugene, OR, USA) and a Qubit 3.0 Fluorometer (Invitrogen™, Life Technologies Holdings Pte Ltd, Singapore; manufactured in Malaysia). RNA purity was assessed spectrophotometrically based on the A260/A280 and A260/A230 ratios using a Nanodrop 2000 UV Spectrophotometer (Thermo Fisher Scientific, Waltham, MA, USA). Samples yielding sufficient RNA for downstream cDNA synthesis and library preparation were included in the targeted RNA-seq workflow.

### 2.5. Library Preparation and Targeted NGS RNA Sequencing

Targeted transcriptome sequencing was performed using the Ion AmpliSeq™ Transcriptome Human Gene Expression Panel, Chef-Ready Kit (cat. no. A31446; Thermo Fisher Scientific, Carlsbad, CA, USA). This amplicon-based assay measures gene-level expression across more than 20,000 human RefSeq genes using approximately 20,800 primer pairs.

The manufacturer-recommended input of 10 ng total RNA per sample was used for both blood and urine libraries. Total RNA was reverse-transcribed using the SuperScript™ VILO™ cDNA Synthesis Kit (cat. no. 11754050; Invitrogen™, Thermo Fisher Scientific, Carlsbad, CA, USA). Subsequent library preparation on the Ion Chef™ System included multiplex amplification of transcript targets, partial digestion of primer sequences, adapter and barcode ligation, library purification, quantification, and pooling according to the manufacturer’s workflow. Universal Human Reference RNA (cat. no. QS0639; Thermo Fisher Scientific, Waltham, MA, USA) was included as an external RNA control. Prepared libraries were quantified using the Ion Library TaqMan™ Quantitation Kit (cat. no. 4468802; Thermo Fisher Scientific, Vilnius, Lithuania).

Up to eight barcoded libraries were pooled per Ion 540™ chip (cat. no. A27766; Thermo Fisher Scientific, Carlsbad, CA, USA). Automated library preparation, clonal amplification, enrichment, and chip loading were performed using the Ion Chef™ System (Thermo Fisher Scientific, Waltham, MA, USA). Sequencing was carried out on an Ion GeneStudio™ S5 System (Thermo Fisher Scientific, Waltham, MA, USA) using Ion 540™ chips (cat. no. A27766) and the Ion 540™ Kit-Chef (cat. no. A30011; Thermo Fisher Scientific, Carlsbad, CA, USA). The Chef-Ready workflow supports the preparation of up to eight barcoded transcriptome libraries per Ion Chef™ run.

Sequencing data were processed on an Ion Torrent™ Server using Torrent Suite™ Software version 5.18.2 and the ampliSeqRNA plugin version 5.18.0.0 (Thermo Fisher Scientific, Waltham, MA, USA). Primary processing included base calling, quality filtering, barcode demultiplexing, trimming of technical sequences, read alignment, and gene-level quantification. Output files included raw gene-level read counts (.bcmatrix), reads-per-million-normalized expression matrices (.rpm.bcmatrix), and barcode- and run-level quality-control summaries (.bc_summary). Chip, sequencing run, barcode, and study-group metadata were retained for downstream quality control, integration of count matrices, and assessment of technical variability.

### 2.6. Differential Expression Analysis

Differential gene expression analysis was performed in R version 4.6.0 [38] using DESeq2 version 1.52.0 [39]. Raw gene-level read counts exported from Ion AmpliSeq™ RNA.bcmatrix files were used for statistical modeling. Reads-per-million-normalized matrices (.rpm.bcmatrix) were used only for preliminary exploratory visualization and quality assessment and were not used for differential expression testing.

Count matrices generated from individual sequencing chips and runs were merged into a single gene-by-sample matrix. Sample identifiers were standardized and matched to clinical and technical metadata, including biofluid type, disease condition, sequencing chip, sequencing run, patient identity, and available clinical variables. For the final urine and paired blood–urine analyses, technical ERCC spike-in features, identified by the “ERCC-“ prefix, were removed prior to statistical analysis, whereas endogenous human genes belonging to the ERCC gene family were retained.

For the independent sepsis-versus-control comparisons, library-size normalization was performed using the default DESeq2 median-of-ratios method. Gene prefiltering was applied separately after defining the sample subset for each analysis. For the primary blood and urine comparisons, genes with at least 10 raw counts in at least three samples were retained. Differential expression was assessed using Wald tests implemented in DESeq2. Independent filtering was applied with α = 0.05, and *p* values were adjusted for multiple testing using the Benjamini–Hochberg procedure [40]. For the main categorical comparisons, differentially expressed genes (DEGs) were defined as those with an adjusted *p* value < 0.05 and an absolute log2 fold change (|log2FC|) ≥ 1.

For the independent sepsis-versus-control analyses, the primary DESeq2 model included disease condition as the explanatory factor (~condition). Blood samples from patients with sepsis were compared with blood samples from controls using the sepsis-versus-control contrast. As a sensitivity analysis, a batch-adjusted blood model additionally included sequencing batch, derived from sequencing chip/run metadata (~batch + condition). Concordance between the primary and batch-adjusted models was assessed based on the direction, magnitude, and correlation of their log2FC estimates. Robust core DEGs were defined as genes that met the DEG criteria in both models and exhibited the same direction of effect.

Because sex distribution differed between the blood sepsis and control groups, with a higher proportion of women among controls, differential expression of sex-linked genes could reflect sex-related confounding rather than sepsis-associated biology. Inspection of genes showing the largest discrepancies in log2FC estimates between the primary and batch-adjusted blood models identified several sex-linked genes. Therefore, as a secondary quality-control step, a curated set of sex-linked markers (*EIF1AY*, *KDM5D*, *DDX3Y*, *ZFY*, *TTTY15*, *USP9Y*, *RPS4Y1*, *UTY*, *SRY*, *TMSB4Y*, *NLGN4Y*, *TXLNGY*, *XIST*, and *TSIX*) was excluded, when present, from the blood robust-core DEG lists used for downstream over-representation analysis and selected visualizations. This filtering did not affect DESeq2 model fitting, the complete differential expression results, or the complete ranked gene lists used for gene set enrichment analysis.

Urine samples from patients with sepsis were compared with urine samples from controls using the final ERCC spike-in-removed dataset and the “model ~condition” specification. Because the dispersion–mean trend in the urine dataset required local fitting, the model was fitted using ‘fitType = “local”’. A batch-adjusted urine sepsis-versus-control model was not used for inference because sequencing batch/chip and disease condition were substantially confounded.

For the paired blood–urine comparison among patients with sepsis, only complete blood–urine pairs from the same patients were included. Patient identity was incorporated as a blocking factor and biofluid as the factor of interest in the DESeq2 model (~patient_id + biofluid). Urine was designated as the reference level, and the contrast compared blood to urine. Genes with a sum of at least 10 raw counts across all samples in the paired dataset were retained. Size factors were estimated using sfType = “poscounts”. Accordingly, positive log2FC values indicated higher expression in blood, whereas negative values indicated higher expression in urine.

Associations between urinary gene expression and serum creatinine were evaluated using separate categorical and continuous DESeq2 analyses, each with analysis-specific model specifications, gene prefiltering criteria, and effect-size thresholds, as detailed in Section 2.9.

Variance-stabilized expression values were used for exploratory visualization, including principal component analysis (PCA), sample-level correlation analysis, heat maps, box plots, and scatter plots, as described in Section 2.8.

### 2.7. Functional Enrichment and Gene Set Enrichment Analysis

Functional enrichment analyses were performed in R version 4.6.0 [38] using clusterProfiler version 4.20.0 [41], fgsea version 1.38.0 [42], org.Hs.eg.db version 3.23.1, AnnotationDbi version 1.74.0, msigdbr version 26.1.0, and rWikiPathways version 1.32.0. Over-representation analysis (ORA) was applied to DEG lists derived from the blood and urine sepsis-versus-control analyses, the paired blood–urine comparison, and the serum creatinine analyses. Gene set enrichment analysis (GSEA) was applied to ranked gene lists from the blood, urine, and categorical and continuous serum creatinine models.

For ORA, genes with positive and negative effect estimates were analyzed separately according to the direction of the corresponding contrast or continuous-model coefficient. DEG definitions for the main categorical comparisons followed the criteria specified in Section 2.6, whereas analysis-specific criteria for the serum creatinine models are described in Section 2.9. Where available, robust-core DEG sets retained across the corresponding primary and batch-adjusted models were additionally analyzed. For blood ORA, the robust-core DEG lists were further filtered to exclude the sex-linked marker genes described in Section 2.6, thereby reducing the contribution of direct sex-linked signals associated with the unequal sex distribution between the study groups. This additional filtering was not applied to the complete ranked gene lists used for GSEA. For Gene Ontology (GO) and KEGG analyses, gene symbols were mapped to Entrez Gene identifiers using AnnotationDbi version 1.74.0 and org.Hs.eg.db version 3.23.1; genes without valid mappings were excluded.

ORA was performed using the GO Biological Process [43], KEGG *Homo sapiens* [44], and WikiPathways gene sets [45]. KEGG pathway annotations were retrieved online through clusterProfiler::enrichKEGG version 4.20.0 during analyses conducted between 15 June and 22 July 2026. For the urine, paired blood–urine, and serum creatinine GO and KEGG analyses, the background universe was restricted to genes retained for statistical testing and successfully mapped to Entrez Gene identifiers. WikiPathways analyses based on a locally stored transposed gene matrix file used the intersection of the tested genes and the genes represented in the corresponding collection. The blood robust-core ORA and WikiPathways analyses performed using enrichWP clusterProfiler version 4.20.0 used the default annotation universe of the respective enrichment function. WikiPathways analyses used either rWikiPathways or a locally stored *Homo sapiens* WikiPathways release dated 10 June 2026, depending on the analysis. Multiple-testing correction was performed separately for each database and direction using the Benjamini–Hochberg procedure [40], and terms with an adjusted *p* value < 0.05 were considered statistically significant.

GSEA was performed without applying a prior significance or effect-size threshold for DEGs [42]. Genes with finite DESeq2 Wald statistics were ranked in descending order of the test statistic. For categorical comparisons, positive statistics indicated higher expression in the numerator group of the specified contrast, whereas negative statistics indicated higher expression in the reference group. For continuous serum creatinine models, positive statistics indicated increasing expression with increasing creatinine, whereas negative statistics indicated decreasing expression.

The 50 Hallmark gene sets from the Molecular Signatures Database (MSigDB release 2026.1.Hs) [46] were obtained using msigdbr version 26.1.0. GSEA was performed using clusterProfiler::GSEA clusterProfiler version 4.20.0 with the multilevel method; the final continuous-creatinine GSEA was performed using fgsea::fgseaMultilevel fgsea version 1.38.0. Gene sets containing between 10 and 500 genes after intersection with the ranked gene list were tested. No nominal *p* value prefiltering was applied (pvalueCutoff = 1 in the clusterProfiler analyses), and eps = 0 was used to improve the estimation of small *p*-values. Fixed random seeds were used to ensure reproducibility.

GSEA results were summarized using the enrichment score, normalized enrichment score (NES), nominal *p* value, and Benjamini–Hochberg-adjusted *p* value [40]. The adjusted *p* value was interpreted as the false discovery rate (FDR). Positive and negative NES values were interpreted based on the direction of the underlying ranked statistic. Results with an FDR < 0.25 were considered exploratory, whereas an FDR < 0.05 was reported as a more stringent threshold.

### 2.8. Data Visualization and Figure Generation

Data visualization and figure generation were performed in R version 4.6.0 [38] using ggplot2 version 4.0.3 [47], ggrepel version 0.9.8, pheatmap version 1.0.13, EnhancedVolcano version 1.30.0, enrichplot version 1.32.0, scales version 1.4.0, patchwork version 1.3.2, and tidyverse version 2.0.0.

DESeq2-normalized counts and variance-stabilized expression values were used for exploratory visualization, clustering, and presentation of gene expression patterns. Statistical inference was based exclusively on raw count data, as described in Section 2.6. Variance-stabilizing transformation was performed using DESeq2. The transformation was applied with ‘blind = FALSE’ for the main categorical and paired comparisons and with ‘blind = TRUE’ for the continuous serum creatinine analyses.

PCA was performed on variance-stabilized expression values using either the DESeq2 ‘plotPCA’ function or principal component decomposition with ‘prcomp’, depending on the analysis. PCA plots were used descriptively to assess the overall structure of the transcriptomic data, sample separation by disease condition or biofluid, variation associated with serum creatinine, and potential technical structure related to sequencing batch or chip. Sample-to-sample distance and correlation heatmaps were additionally used for exploratory quality assessment.

Volcano plots were generated using ggplot2 version 4.0.3 or EnhancedVolcano version 1.30.0. The horizontal axis represented the estimated log2 fold change, and the vertical axis represented −log10 of the Benjamini–Hochberg-adjusted *p* value [40]. Genes were classified according to the statistical and effect-size thresholds defined for the corresponding analysis in Section 2.6 and Section 2.9. Selected genes were labeled using ggrepel version 0.9.8 based on statistical ranking and, where indicated, biological interpretability. Gene labeling was performed for visualization purposes only and did not affect statistical testing or DEG classification.

Heatmaps were generated for top-ranked DEGs, robust core DEGs, or explicitly curated gene sets relevant to the corresponding analysis. Variance-stabilized or DESeq2-normalized expression values were used as indicated, with row-wise standardization applied where appropriate to display relative expression patterns across samples. For the principal DEG heatmaps, hierarchical clustering generally used correlation distance and complete linkage. Euclidean distances were used for selected sample-distance heatmaps. In figures designed for presentation with a predefined sample or gene order, clustering was constrained or disabled.

Boxplots were used to display the expression of representative genes across study groups or biofluids. Scatterplots were used to visualize associations between serum creatinine and variance-stabilized expression values or transcriptomic module scores. Least-squares trend lines were added for descriptive purposes, with confidence intervals shown where applicable. For continuous creatinine analyses, serum creatinine was plotted on a logarithmic scale or modeled as log2-transformed creatinine, as specified in Section 2.9. Statistical inference for these associations was based on the corresponding DESeq2 models rather than on the plotted regression lines.

ORA and GSEA results were visualized using bar plots, dot plots, NES plots, and enrichment curves. Terms or pathways were displayed according to their adjusted *p* values, FDR, NES, and biological interpretability, with the number of displayed terms limited for readability. Multi-panel figures were assembled using patchwork and exported in high-resolution raster and vector formats.

### 2.9. Serum Creatinine-Associated Transcriptomic Analyses

Associations between gene expression in the urinary cellular fraction and serum creatinine were evaluated in an exploratory analysis restricted to urine samples from patients with sepsis for whom both RNA-seq data and serum creatinine measurements were available in the linked clinical metadata. Serum creatinine was treated as a continuous clinical measure related to renal function rather than as a direct molecular measure of kidney injury or an independently validated diagnostic or prognostic biomarker. Serum creatinine concentrations were measured using the Jaffé method on a BA400 automated clinical chemistry analyzer with the Creatinine reagent (REF 21502; BioSystems S.A., Barcelona, Spain).

Serum creatinine was evaluated using two complementary parameterizations. In the categorical analysis, patients were classified as having high (>110 μmol/L) or low (≤110 μmol/L) serum creatinine. This study-defined cutoff was selected with reference to the 110 μmol/L boundary of the renal component of the Sequential Organ Failure Assessment (SOFA) score. The primary DESeq2 model included creatinine group as the explanatory factor (~creatinine_group), with the low-creatinine group as the reference and a high-versus-low contrast. A batch-adjusted sensitivity model additionally included sequencing chip (~sequencing_chip + creatinine_group) after confirmation that the model matrix was of full rank.

Before fitting the categorical models, genes with at least 10 raw counts in at least four of the 21 included urine samples were retained. Strict DEGs were defined by an adjusted *p* value < 0.05 and |log2FC| ≥ 1. A secondary relaxed criterion of an adjusted *p* value < 0.10 and |log2FC| ≥ 0.58 was used for sensitivity analyses. Positive log2FC values indicated higher expression in the high-creatinine group, whereas negative values indicated higher expression in the low-creatinine group.

In the continuous analysis, serum creatinine values were log2-transformed and subsequently standardized to a mean of zero and a standard deviation of one. The primary model was specified as ~creatinine_log2_z, and the batch-adjusted model additionally included sequencing chip (~chip_id + creatinine_log2_z). The adjusted model was fitted only after confirmation that the model matrix was of full rank and used fitType = “local”. Genes with at least five raw counts in at least three samples were retained.

For the continuous models, the DESeq2 coefficient β represented the log2 expression change associated with a one-standard-deviation increase in log2-transformed serum creatinine. The primary strict criterion was an adjusted *p* value < 0.05 and |β| ≥ 0.58, corresponding to an approximately 1.5-fold expression change per standard-deviation increase in log2-transformed creatinine. A more conservative criterion of an adjusted *p* value < 0.05 and |β| ≥ 1 and a relaxed criterion of an adjusted *p* value < 0.10 and |β| ≥ 0.38 were additionally evaluated. Positive coefficients indicated increasing expression with increasing serum creatinine, whereas negative coefficients indicated decreasing expression.

For both categorical and continuous analyses, robust core DEGs were defined as genes that met the corresponding strict criterion in both the primary and batch-adjusted models and retained the same direction of effect. Direction-specific ORA and Hallmark GSEA were performed as described in Section 2.7. Robust Hallmark pathways were defined as pathways with FDR < 0.25 in both the primary and batch-adjusted models and a concordant NES direction. Pathways meeting FDR < 0.05 in both models were additionally reported as a stringent robust subset.

To assess the influence of the highest serum creatinine value, the continuous analysis was repeated after excluding the sample with the maximum concentration (1144 μmol/L; *n* = 20). For direct coefficient comparability, log2-transformed creatinine values were standardized using the mean and standard deviation from the full 21-sample cohort. Unadjusted and chip-adjusted DESeq2 models were refitted using the original filtering and DEG criteria. Robustness was assessed by gene overlap and directional concordance, correlations of coefficients and Wald statistics, persistence of robust-core genes, and concordance of Hallmark NES values.

Potential demographic confounding was evaluated by refitting the continuous model with adjustment for sex (available for all 21 patients), age (*n* = 20), and both covariates. Given the small subgroup size, separate sex-stratified genome-wide analyses were not performed; instead, an exploratory sex-by-creatinine interaction model was fitted, with interaction effects evaluated using Benjamini–Hochberg-adjusted *p* values.

To determine whether the urinary creatinine-associated transcriptional pattern was paralleled by a systemic whole-blood response, an exploratory matched cross-biofluid analysis was performed in the same 21 patients with paired blood and urine RNA-seq profiles and serum creatinine measurements. Serum creatinine was transformed and standardized identically in both biofluids. For blood, continuous models were specified as ~creatinine_log2_z and ~sequencing_batch + creatinine_log2_z; for urine, the corresponding models were ~creatinine_log2_z and ~sequencing_chip + creatinine_log2_z. Genes with at least five raw counts in at least three samples were retained within each biofluid. Direct gene-level comparison was restricted to genes tested in both adjusted models. Cross-biofluid concordance was evaluated using Pearson and Spearman correlations of DESeq2 coefficients and Wald statistics, overlap and directional concordance of strict DEGs and robust-core genes, and concordance of Hallmark NES values. The same strict DEG and robust-core definitions used for the continuous urinary analysis were applied. Robust Hallmark pathways were defined independently within each biofluid as pathways with FDR < 0.25 in both the primary and sequencing-adjusted models and concordant NES direction.

Priority transcripts for orthogonal validation were selected using a predefined multistage procedure. Candidates had to belong to the original continuous-creatinine robust core, remain supported after exclusion of the highest-creatinine sample, retain significance and direction in sex-, age-, and sex-plus-age-adjusted models, show no significant sex-by-creatinine interaction (FDR < 0.05), have baseMean ≥10, and be supported by the categorical creatinine analysis. Technical feasibility required raw counts ≥1 in ≥80% of samples (≥17/21), counts ≥5 in ≥2/3 (≥14/21), and a median raw count ≥5. The final panel comprised the five highest-ranked positively and five highest-ranked negatively associated transcripts meeting all criteria, with no manual substitutions.

All serum creatinine-associated transcriptomic analyses were considered exploratory. Owing to the observational study design and the limited size of the urine subgroup, these analyses were not used to derive causal, diagnostic, or prognostic models. Complete SOFA scores, formal KDIGO AKI classification, serial urine-output data, and vasopressor exposure were not available for the urinary RNA-seq cohort and therefore could not be included in the analysis.

### 2.10. Data and Code Availability

De-identified processed gene-expression data and associated non-sensitive sample metadata generated in this study have been deposited in the NCBI Gene Expression Omnibus (GEO) under accession GSE345856. Raw sequencing data have been deposited in the corresponding NCBI Sequence Read Archive (SRA) records. These records are currently available for peer review through private-access mechanisms and will be released publicly in accordance with NCBI policies.

The R scripts used for data preprocessing, differential expression analysis, functional enrichment, gene set enrichment analysis, and figure generation are publicly available in the GitHub repository Targeted Transcriptomic Analysis of Blood and Urine in Sepsis (https://github.com/beloslav1976-svg/targeted-sepsis-transcriptomics, accessed on 6 October 2026). The version used for the present manuscript, release v1.1.0, is permanently archived in Zenodo (https://doi.org/10.5281/zenodo.22096555). The repository includes workflow documentation, an R package lockfile, analysis parameters, pipeline manifests, and script- and output-level provenance records. The code repositories contain no participant-level clinical data or direct participant identifiers.

## 3. Results

### 3.1. Study Cohort, Sample Selection, and RNA-Seq Analysis Workflow

A total of 100 adults with suspected or clinically diagnosed sepsis were screened. Biological specimens were collected from 65 patients, of whom 35 met the biospecimen, clinical-annotation, and laboratory quality requirements for inclusion in the final RNA-seq cohort. The control group comprised 10 healthy adult volunteers.

Whole-blood RNA-seq was performed for 35 patients with sepsis and 10 controls. Urinary cellular-fraction RNA-seq was performed for 21 patients with sepsis and 7 controls whose samples met the RNA quantity and quality requirements. Paired blood–urine RNA-seq profiles were therefore available for 21 patients with sepsis and 7 controls.

Among patients with sepsis, 24/35 (68.6%) were male. Age was available for 34/35 patients, with a median of 63.5 years (IQR, 51.3–69.8; range, 25–88). The control group included 2 men and 8 women and had a median age of 44.5 years (IQR, 39.8–56.8; range, 25–66). Thus, the sepsis and control groups differed in both age and sex distribution.

In-hospital mortality among patients with sepsis was 51.4% (18/35), mechanical ventilation was required in 18/35 patients (51.4%), and microbiological cultures were positive in 13/35 patients (37.1%). Median serum creatinine was 133.0 µmol/L (IQR, 69.7–225.3; range, 40.4–1144.0), and concentrations exceeded 110 µmol/L in 20/35 patients (57.1%). Among the 21 patients with urinary RNA-seq data used for the creatinine-associated transcriptomic analyses, 12 had serum creatinine >110 µmol/L and 9 had concentrations ≤110 µmol/L.

**Figure 1:**
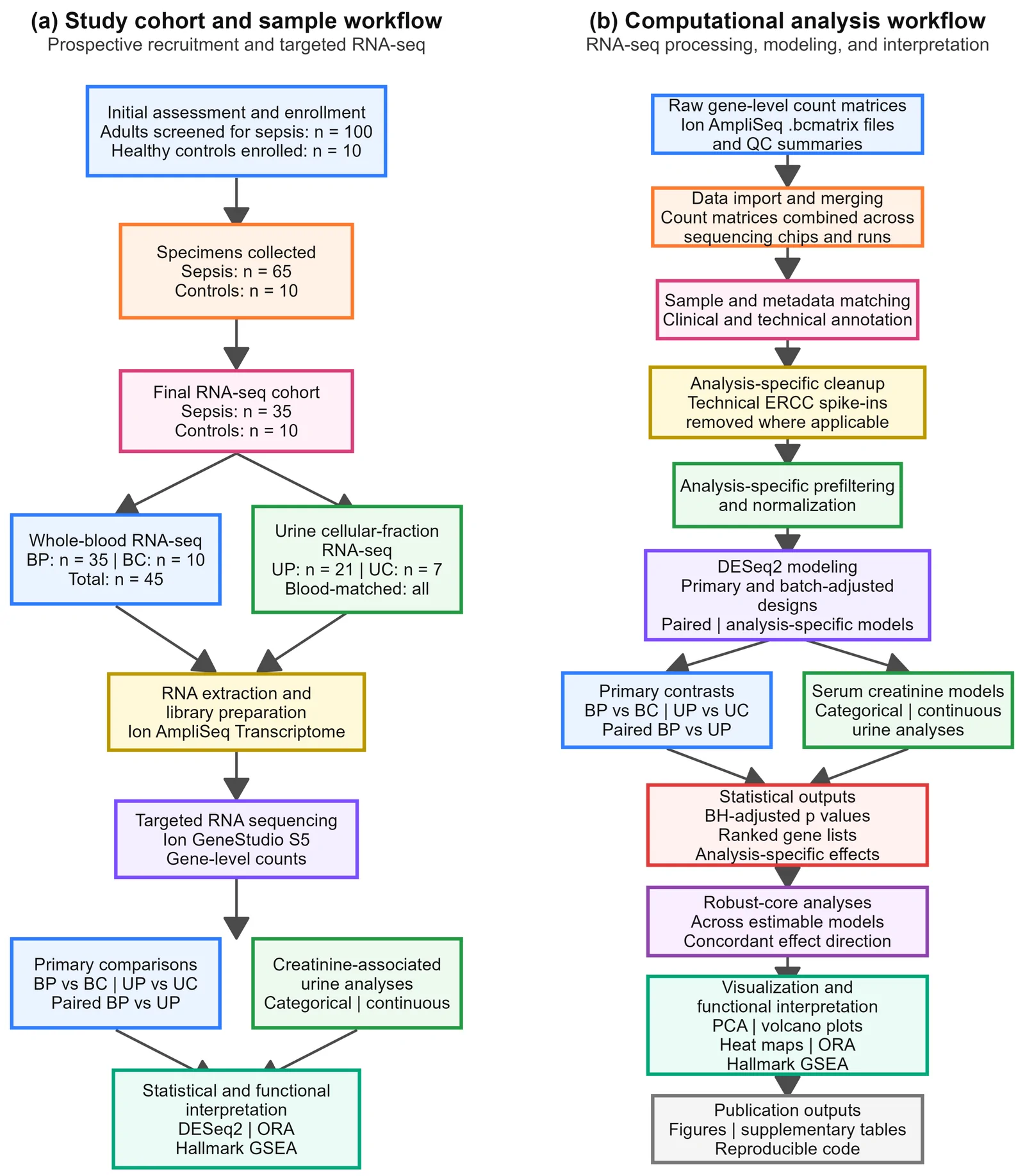
Study cohort, sample selection, and targeted RNA-seq analysis workflow. (**a**) Of 100 adults screened for suspected or clinically diagnosed sepsis, biological specimens were collected from 65, and 35 met the biospecimen, clinical annotation, and laboratory quality requirements for inclusion in the final RNA-seq cohort. Ten healthy controls were included. Whole-blood RNA-seq comprised 35 sepsis (BP) and 10 control (BC) samples, whereas urinary cellular-fraction RNA-seq comprised 21 sepsis (UP) and 7 control (UC) samples. Each participant with an available urine RNA-seq profile also had a matched blood RNA-seq profile. (**b**) Raw gene-level count matrices and quality-control summaries were merged across sequencing chips and runs, linked to clinical and technical metadata, subjected to analysis-specific feature cleanup and gene prefiltering, and analyzed using DESeq2. Downstream analyses included differential expression testing, ranked-gene analysis, robust-core assessment where estimable, visualization, over-representation analysis, Hallmark gene set enrichment analysis, and categorical and continuous serum creatinine-associated analyses. BC, blood control; BH, Benjamini–Hochberg; BP, blood sepsis; GSEA, Gene Set Enrichment Analysis; ORA, over-representation analysis; PCA, principal component analysis; UC, urine control; UP, urine sepsis.

Cohort selection and the analytical workflow are summarized in Figure 1. Clinical characteristics and sample availability are presented in Table 1, and de-identified sample-level annotations are provided in Supplementary Table S1.

### 3.2. Biofluid-Specific and Shared Transcriptomic Programs in Sepsis

#### 3.2.1. Blood Transcriptomics Reveals Systemic Myeloid Activation and Suppression of Adaptive Immune Programs

**Figure 2:**
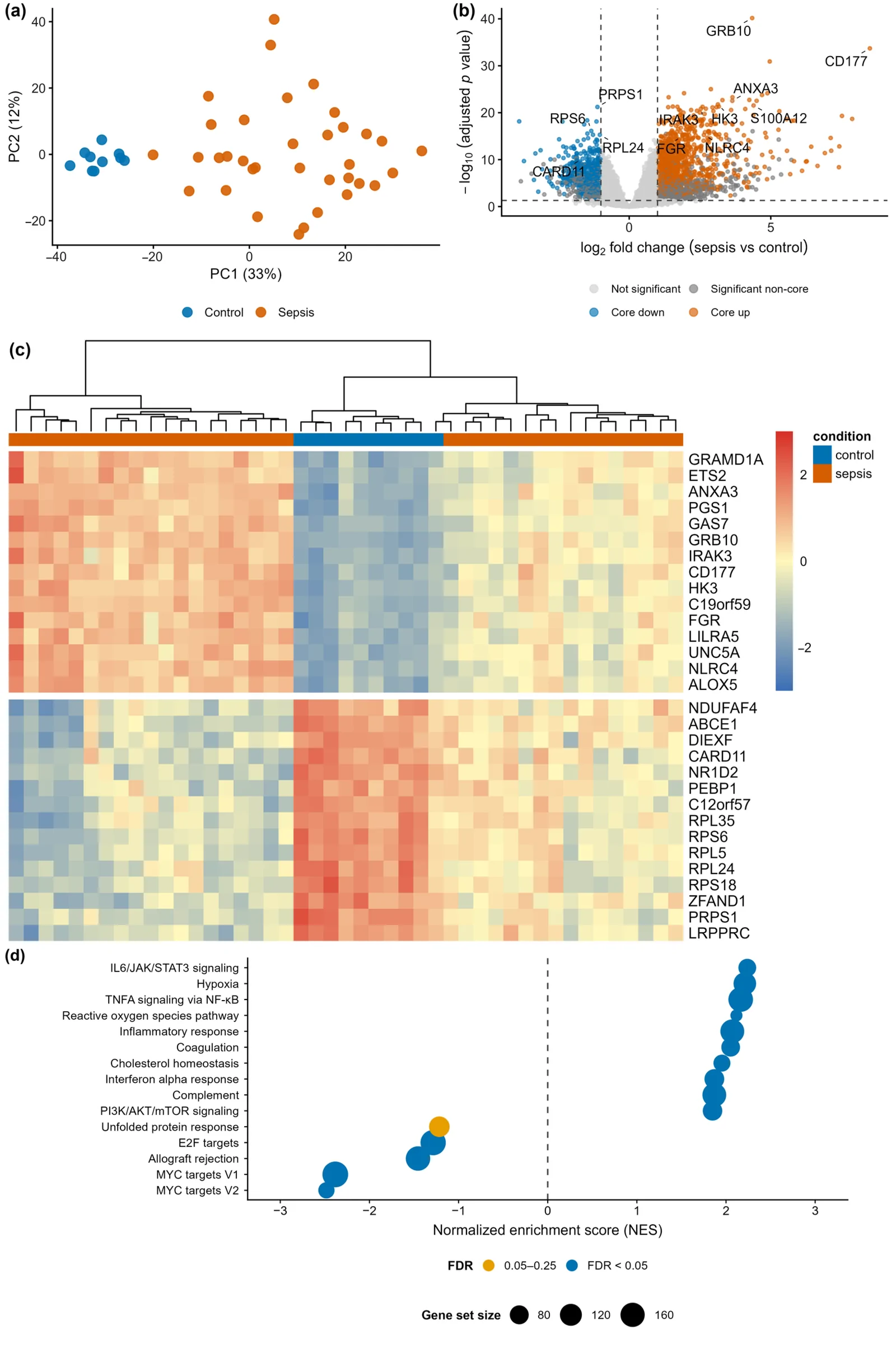
Blood transcriptomics reveals systemic myeloid activation and suppression of adaptive immune programs in sepsis. (**a**) PCA of variance-stabilized blood RNA-seq expression profiles from patients with sepsis (*n* = 35) and healthy controls (*n* = 10). PC1 and PC2 explained 33% and 12% of the total variance, respectively. (**b**) Volcano plot for the primary blood sepsis-versus-control DESeq2 model. Robust-core DEGs met the adjusted *p* value < 0.05 and |log2FC| ≥ 1 criteria in both the primary and batch-adjusted models and had concordant effect directions. Values of −log10 (adjusted *p* value) were capped at 45 for display. (**c**) Heatmap of 30 representative robust-core DEGs (15 upregulated and 15 downregulated), selected based on batch-adjusted statistical significance and biological relevance. Rows show row-scaled variance-stabilized expression, and columns represent samples, with disease condition indicated by the top annotation bar. Genes were ordered by Euclidean-distance complete-linkage clustering within the upregulated and downregulated groups, which are separated by a horizontal gap; samples were clustered using the same method. (**d**) Selected Hallmark gene sets with FDR < 0.25 from preranked GSEA using the complete DESeq2 Wald-statistic-ranked gene list. Positive normalized enrichment scores (NESs) indicate enrichment in sepsis blood, whereas negative NES indicate relative enrichment in control blood. Point size represents gene set size, and color represents the FDR category. FDR, false discovery rate; GSEA, gene set enrichment analysis; NES, normalized enrichment score.

Blood transcriptomic analysis included 45 samples from 35 patients with sepsis and 10 healthy controls; 12,400 genes were retained after prefiltering. PCA of variance-stabilized expression values separated the groups primarily along PC1, which accounted for 33% of the total variance (Figure 2a). Controls formed a compact cluster, whereas sepsis samples showed greater transcriptomic heterogeneity. PCA colored by sequencing batch is shown in Figure S1a.

The primary DESeq2 model identified 2659 DEGs at an adjusted *p* value < 0.05 and an absolute |log2FC| ≥ 1, including 1660 upregulated and 999 downregulated genes in sepsis blood. The batch-adjusted sensitivity model identified 4125 DEGs, including 2093 upregulated and 2032 downregulated genes. Despite the difference in DEG counts, effect estimates were concordant between models (Pearson r = 0.815; Spearman ρ = 0.859). Model diagnostics and log2FC concordance are shown in Figure S1b–e.

The robust blood core comprised 1796 genes that met the DEG criteria in both models and had concordant effect directions, including 1133 upregulated and 663 downregulated genes (Figure 2b; Tables S2 and S3). None of the prespecified sex-linked genes examined in the sensitivity analysis was present in the robust core (Table S4).

A heatmap of 30 representative robust-core DEGs, including 15 upregulated and 15 downregulated genes, largely separated sepsis and control samples (Figure 2c). Genes upregulated in sepsis included *CD177*, *ANXA3*, *HK3*, *IRAK3*, *FGR*, *NLRC4*, *ALOX5*, and *GRB10*, consistent with neutrophil–myeloid activation and inflammatory metabolic remodeling. Downregulated genes included *CARD11*, *RPS6*, *RPS18*, *RPL24*, *RPL5*, and *PRPS1*, consistent with attenuation of adaptive immune and translational programs.

ORA identified opposing functional programs within the robust core. Upregulated genes were enriched for myeloid leukocyte activation, inflammatory and innate immune responses, cytokine and interleukin-1 production, phagocytosis, and responses to lipopolysaccharide. Downregulated genes were enriched for lymphocyte differentiation and activation, antigen receptor signaling, and T-cell receptor signaling (Figure S1f). KEGG and WikiPathways analyses showed concordant enrichment of phagocytic, TNF, NOD-like receptor, MYD88, chemokine, and complement pathways among upregulated genes and ribosomal and T-cell-associated pathways among downregulated genes (Figure S1g,h; Table S5).

Hallmark GSEA of the complete Wald-statistic-ranked gene list confirmed this threshold-independent pattern (Figure 2d). Among 50 Hallmark gene sets, 33 showed positive enrichment in sepsis blood at a FDR < 0.25, including 26 at FDR < 0.05. Prominent positively enriched programs included IL-6/JAK/STAT3 signaling, hypoxia, TNF-α signaling via NF-κB, inflammatory response, reactive oxygen species, coagulation, complement, interferon-α response, and PI3K/AKT/mTOR signaling. Five gene sets showed negative enrichment at FDR < 0.25, including four at FDR < 0.05: MYC targets V1 and V2, allograft rejection, E2F targets, and the unfolded protein response (Table S6).

Together, these findings indicate a systemic shift toward myeloid–neutrophil inflammatory activation accompanied by suppression of adaptive immune and translational programs in sepsis blood.

#### 3.2.2. Exploratory Urine RNA-Seq Identifies Inflammatory and Epithelial–Barrier-Related Expression Patterns in Sepsis

**Figure 3:**
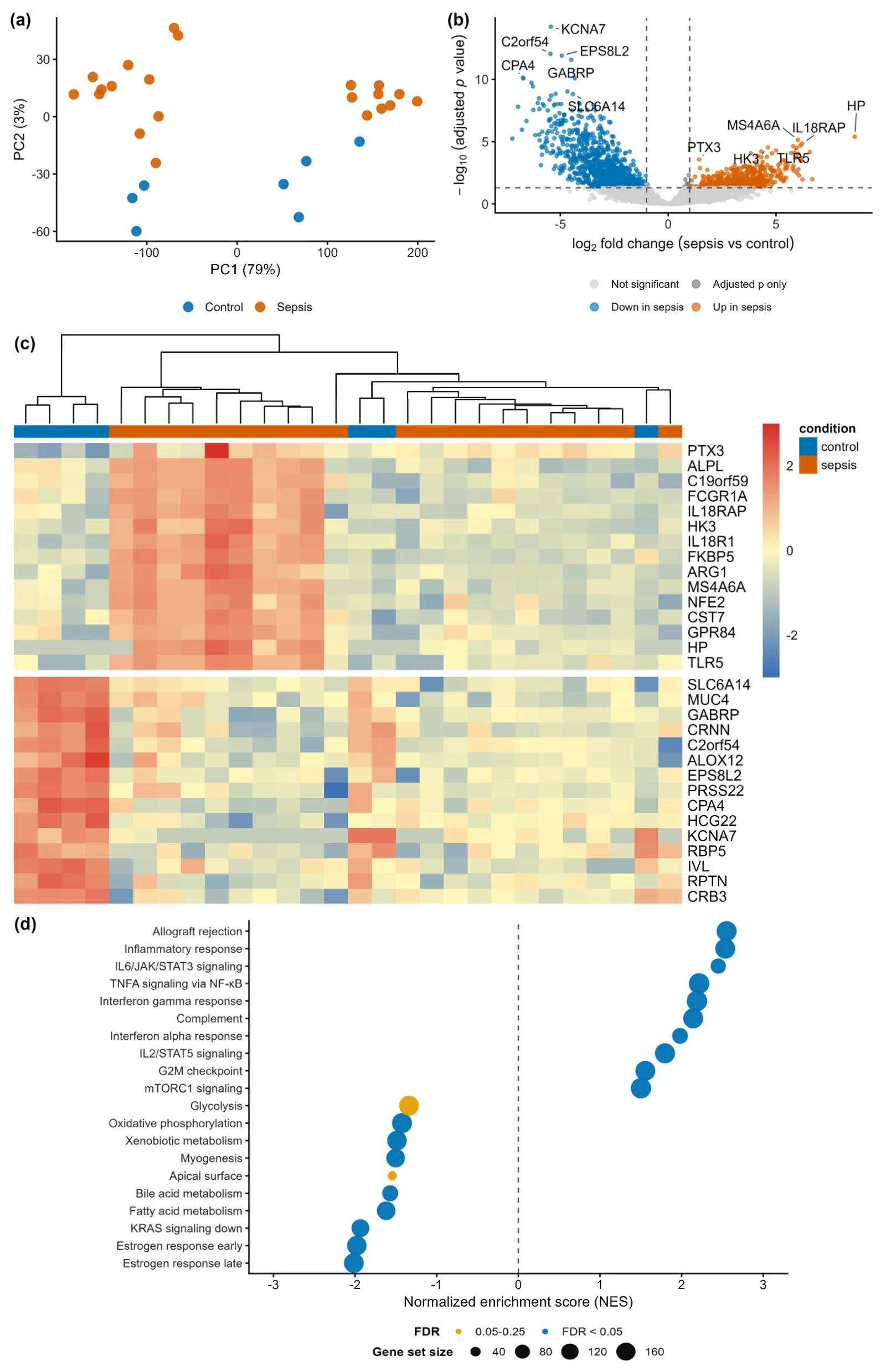
Exploratory urine RNA-seq identifies inflammatory and epithelial-barrier-related expression patterns in sepsis. (**a**) PCA of variance-stabilized RNA-seq expression profiles of the urinary cellular fraction from patients with sepsis (*n* = 21) and healthy controls (*n* = 7). PC1 and PC2 explained 79% and 3% of the total variance, respectively. (**b**) Volcano plot for the urine sepsis-versus-control DESeq2 model. DEGs were defined by an adjusted *p* value < 0.05 and |log2FC| ≥ 1; 587 genes were upregulated and 965 were downregulated in sepsis. Values of −log10 (adjusted *p* value) were capped at 20 for visualization. (**c**) Heatmap of 30 representative DEGs, comprising 15 upregulated and 15 downregulated genes selected on the basis of statistical significance and biological relevance. Rows show row-scaled variance-stabilized expression, and columns represent samples. Genes were ordered by Euclidean-distance complete-linkage clustering within the upregulated and downregulated groups, which are separated by a horizontal gap; samples were clustered using the same method. (**d**) Selected Hallmark gene sets with FDR < 0.25 from pre-ranked GSEA using the complete DESeq2 Wald-statistic-ranked gene list. NES indicate enrichment in sepsis urine, whereas negative NES indicate relative enrichment in control urine. Point size represents gene set size, and color indicates the FDR category. Because sequencing chip and disease condition were substantially confounded, all urine sepsis-versus-control results were interpreted as exploratory. FDR, false discovery rate; GSEA, Gene Set Enrichment Analysis; NES, normalized enrichment score.

RNA-seq profiles of the urinary cellular fraction were analyzed in 28 samples from patients with sepsis (*n* = 21) and healthy controls (*n* = 7). PCA of variance-stabilized expression values showed partial separation by disease condition, with PC1 and PC2 explaining 79% and 3% of the total variance, respectively (Figure 3a). Sepsis samples exhibited greater transcriptional heterogeneity than controls. However, sequencing chip and disease condition were substantially confounded (Figure S2a), precluding an interpretable batch-adjusted comparison; therefore, all urine sepsis-versus-control results were considered exploratory.

Among the 16,495 genes retained after prefiltering, 1552 met the differential-expression criteria, including 587 upregulated and 965 downregulated genes in sepsis urine (Figure 3b; Table S7). Model diagnostics, including the MA plot and dispersion estimates, are shown in Figure S2b,c. A heatmap of 30 representative DEGs showed broad separation between sepsis and control samples, together with marked heterogeneity within the sepsis group (Figure 3c). Upregulated genes included *HP*, *MS4A6A*, *IL18RAP*, *TLR5*, *HK3*, *FCGR1A*, *ARG1*, *GPR84*, and *PTX3*, consistent with inflammatory and myeloid activation. Downregulated genes included *KCNA7*, *EPS8L2*, *GABRP*, *CPA4*, *SLC6A14*, *IVL*, *RPTN*, *CRNN*, and *MUC4*, several of which are associated with epithelial, mucosal, or barrier-related functions.

ORA identified opposing functional patterns (Figure S2d–f; Table S8). Upregulated genes were enriched for myeloid leukocyte activation, inflammatory responses, leukocyte migration and chemotaxis, phagocytosis, IL-1β production, neutrophil extracellular trap formation, and chemokine, NOD-like receptor, Toll-like receptor, complement, and prostaglandin signaling. Downregulated genes were enriched for epidermal and epithelial differentiation, tight junctions, aldosterone-regulated sodium reabsorption, peroxisomal function, and retinoid, amino acid, and xenobiotic metabolism. Pathways carrying infection-specific database labels were interpreted as host-response modules rather than evidence of a particular infectious agent.

Pre-ranked Hallmark GSEA using the complete DESeq2 Wald-statistic-ranked gene list supported this pattern without applying a DEG threshold (Figure 3d; Table S9). At FDR < 0.25, 19 gene sets had positive NES values and 13 had negative NES values; 15 and 8 of these, respectively, also met FDR < 0.05. Positively enriched programs included inflammatory response, interferon responses, complement, IL6/JAK/STAT3, TNFA/NF-κB, IL2/STAT5, and mTORC1 signaling. Negative NES values were observed for oxidative phosphorylation, glycolysis, fatty acid, bile acid, and xenobiotic metabolism, and apical-surface programs. Thus, the exploratory urinary transcriptomic profile was characterized by inflammatory–myeloid enrichment together with lower representation of epithelial, barrier, and metabolic programs.

#### 3.2.3. Blood–Urine Comparison Identifies a Shared Inflammatory Core and Extensive Biofluid-Specific Expression Differences

**Figure 4:**
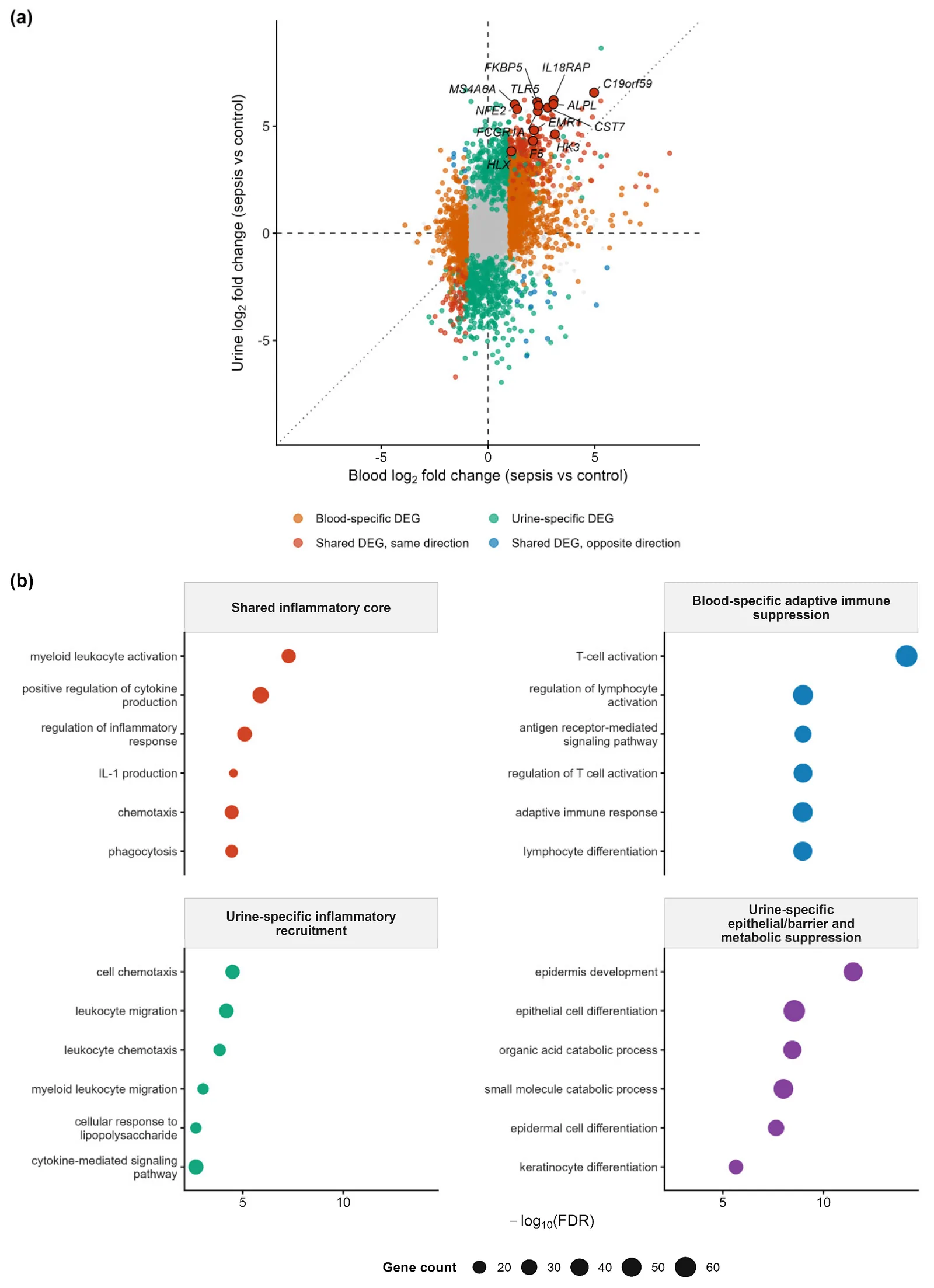
Cross-biofluid comparison identifies a shared inflammatory core and distinct blood and urine expression patterns. (**a**) Comparison of sepsis-versus-control log2 fold-change estimates for 11,849 genes with estimates available in both blood and urine analyses. Blood classifications were based on robust-core DEGs and urine classifications on the primary urine DEGs. Within this common measured-gene universe, 2445 genes were differentially expressed in at least one biofluid: 1365 were blood-specific, 744 were urine-specific, 314 were shared with concordant effect directions, and 22 were shared with discordant directions. Dashed horizontal and vertical lines indicate zero effect, and the dotted diagonal indicates equal effect estimates in both biofluids. Selected representative genes are labelled. (**b**) Selected GO Biological Process terms enriched among concordantly upregulated shared genes, blood-specific downregulated genes, urine-specific upregulated genes, and urine-specific downregulated genes. Point position represents −log10(FDR), point size represents gene count, and colour identifies the functional module. Only terms with FDR < 0.05 are shown. DEG, differentially expressed gene; FDR, false discovery rate.

Cross-biofluid comparison of the sepsis-versus-control signatures included 1796 robust-core blood DEGs and 1552 urine DEGs. Of these, 336 were shared, corresponding to 18.7% of blood DEGs and 21.6% of urine DEGs, whereas 1460 and 1216 were blood- and urine-specific, respectively. Most shared DEGs showed concordant effects (314/336, 93.5%): 254 were upregulated and 60 were downregulated in both biofluids, whereas 22 showed discordant directions. For direct effect-size comparison, Figure 4a was restricted to 11,849 genes with estimates available in both analyses. Within this common measured-gene universe, 2445 genes were differentially expressed in at least one biofluid, comprising 1365 blood-specific, 744 urine-specific, 314 concordant shared, and 22 discordant shared DEGs (Figure 4a; Table S10). Because the urine sepsis-versus-control analysis was exploratory, the terms “blood-specific” and “urine-specific” here describe the observed differential-expression patterns and should not be interpreted as validated compartment-specific sepsis effects.

Concordantly upregulated shared genes included *IL18RAP*, *TLR5*, *HK3*, *FCGR1A*, *CST7*, *ALPL*, *EMR1*, *HLX*, *F5*, *MS4A6A*, *NFE2*, *FKBP5*, and *C19orf59* (Figure S3a). These genes were consistent with a shared myeloid-inflammatory response involving IL-18/TLR- and Fc receptor-associated components and inflammatory–metabolic remodeling. Complete shared, concordant, discordant, blood-specific, and urine-specific DEG sets are provided in Table S10.

GO Biological Process enrichment identified four major functional modules (Figure 4b; Table S11). Concordantly upregulated shared DEGs formed a blood–urine inflammatory core associated with myeloid activation, cytokine production, IL-1 production, phagocytosis, and chemotaxis. Blood-specific downregulated DEGs were enriched for T-cell activation, antigen receptor-mediated signaling, adaptive immune responses, and lymphocyte differentiation. Urine-specific upregulated DEGs were associated with leukocyte migration, chemotaxis, cytokine-mediated signaling, and responses to lipopolysaccharide, whereas urine-specific downregulated DEGs were enriched for epithelial-barrier and metabolic processes.

Paired within-patient analysis of 21 matched blood–urine pairs further demonstrated extensive differences in gene expression between the two biofluids. In the DESeq2 model incorporating patient identity as a blocking factor (~patient_id + biofluid), 15,162 of 20,580 tested genes (73.7%) met the DEG criteria; 7740 showed higher expression in blood and 7422 showed higher expression in urine. PCA based on the complete variance-stabilized expression matrix showed strong separation by biofluid, and the paired volcano plot demonstrated extensive blood- and urine-enriched transcriptional programs (Figure S3b,c; Table S12). Because blood and urinary cellular fractions differ intrinsically in cellular composition and tissue origin, these extensive differences were interpreted descriptively and not as evidence of disease-specific transcriptional compartmentalization.

Functional enrichment of the paired results showed that blood-enriched genes were predominantly associated with RNA processing, translation, vesicular and endosomal transport, and immune signaling. Urine-enriched genes were associated with receptor- and GPCR-related transcriptional programs, epithelial- and barrier-related processes, ion transport, and antimicrobial responses (Table S13). Receptor and sensory-related terms were driven largely by olfactory receptor-family genes and were therefore interpreted as a broad receptor-associated transcriptional signature rather than evidence of olfactory function.

Collectively, these analyses identified a concordant inflammatory–myeloid core shared between blood and urine alongside extensive biofluid-specific expression differences. The blood signature was dominated by systemic myeloid activation and suppression of adaptive immune programs, whereas the exploratory urine signature showed enrichment of leukocyte recruitment and chemotactic pathways together with lower representation of epithelial-barrier and metabolic programs. These differences should be interpreted in the context of the distinct cellular composition and biological origin of the two biofluids.

### 3.3. Serum Creatinine-Associated Urinary Transcriptomic Patterns in Sepsis

#### 3.3.1. Creatinine-Based Stratification Identifies a Serum Creatinine-Associated Urinary Transcriptomic Signature

**Figure 5:**
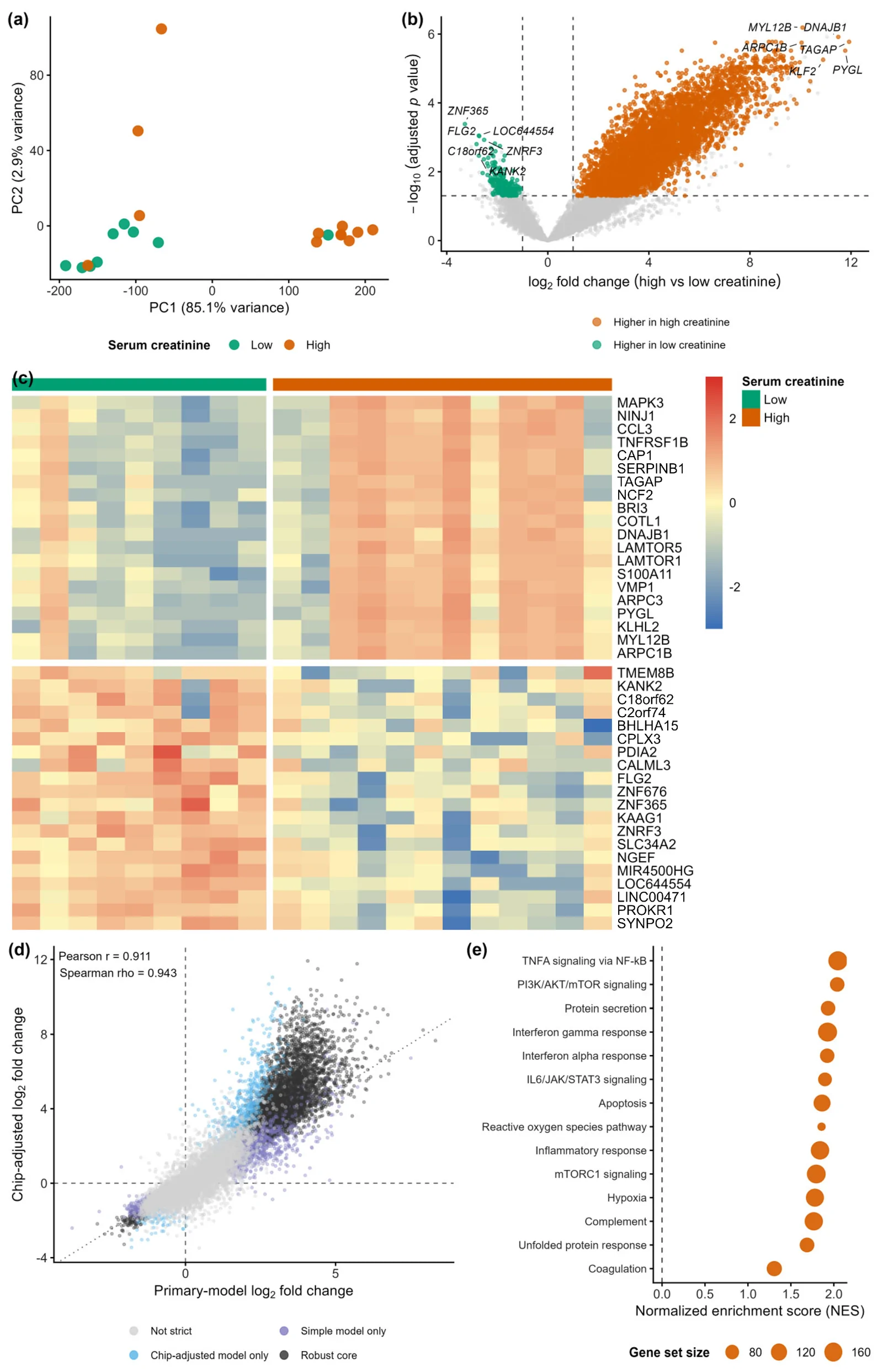
Serum creatinine-associated urinary transcriptomic programs in sepsis. (**a**) PCA of the 500 most variable variance-stabilized genes in urine samples from patients with sepsis stratified by serum creatinine concentration (high, >110 µmol/L, *n* = 12; low, ≤110 µmol/L, *n* = 9). PC1 and PC2 explained 85.1% and 2.9% of the variance, respectively. (**b**) Volcano plot from the sequencing chip-adjusted DESeq2 sensitivity model. Positive log2FC values indicate higher expression in the high-creatinine group. Robust-core DEGs met the adjusted *p* value < 0.05 and |log2FC| ≥ 1 criteria in both the primary and chip-adjusted models and had concordant effect directions. Selected genes are labeled. (**c**) Heatmap of 40 representative robust-core DEGs, comprising 20 genes with higher expression in each creatinine group. Rows show row-scaled variance-stabilized expression values, and columns represent samples ordered by creatinine group and concentration. Genes were clustered separately within each direction using Euclidean distance and complete linkage. (**d**) Comparison of log2FC estimates between the primary and chip-adjusted models for all 15,026 genes with finite estimates (Pearson r = 0.911; Spearman ρ = 0.943). The dotted diagonal denotes equal estimates. (**e**) Selected Hallmark gene sets with FDR < 0.05 from pre-ranked GSEA of the chip-adjusted model. Genes were ranked by the DESeq2 Wald statistic. Positive NES values indicate enrichment in the high-creatinine group; point size represents gene-set size, and color denotes the FDR category. DEG, differentially expressed gene; FDR, false discovery rate; GSEA, Gene Set Enrichment Analysis; NES, normalized enrichment score; PCA, principal component analysis.

To characterize urinary transcriptomic differences associated with serum creatinine, urine samples from 21 patients with sepsis were stratified by serum creatinine concentration: 12 were assigned to the high-creatinine group (>110 µmol/L) and 9 to the low-creatinine group (≤110 µmol/L). PCA of the 500 most variable variance-stabilized genes showed marked transcriptomic separation associated with the creatinine group, with PC1 and PC2 explaining 85.1% and 2.9% of the variance, respectively (Figure 5a). The distribution of samples by sequencing chip is shown in Figure S4a.

The primary DESeq2 model identified 6730 strict DEGs (adjusted *p* value < 0.05 and |log2FC| ≥ 1), including 5900 genes with higher expression in the high-creatinine group and 830 with higher expression in the low-creatinine group. The sequencing chip-adjusted sensitivity model identified 5596 strict DEGs. A robust core of 4990 genes met the DEG criteria in both models and had concordant effect directions, including 4777 genes with higher expression in the high-creatinine group and 213 with higher expression in the low-creatinine group (Table S14). Although the chip-adjusted design matrix was full rank, three sequencing chips contained only high-creatinine samples; the adjusted analysis was therefore interpreted as a sensitivity analysis rather than as complete exclusion of technical confounding.

The chip-adjusted volcano plot showed a strong predominance of genes with higher expression in the high-creatinine group (Figure 5b). Representative genes included *MYL12B*, *DNAJB1*, *TAGAP*, *ARPC1B*, *PYGL*, *KLF2*, *NCF2*, *CCL3*, *S100A11*, and *TNFRSF1B*. Genes with relatively higher expression in the low-creatinine group included *ZNF365*, *FLG2*, *ZNRF3*, *KANK2*, *PDIA2*, *SLC34A1*, and *SLC34A2*. A heatmap of 40 representative robust-core DEGs illustrated opposing high-creatinine-associated inflammatory and stress-response patterns and low-creatinine-associated epithelial and transport-related expression patterns (Figure 5c). Comparison of log2FC estimates between the primary and chip-adjusted models for all 15,026 genes with finite estimates showed high concordance (Pearson r = 0.911; Spearman ρ = 0.943; Figure 5d).

Of the 50 Hallmark gene sets included in the analysis, 49 were successfully evaluated in each model; KRAS signaling down could not be reliably estimated and was retained with missing significance statistics. In the chip-adjusted model, 41 Hallmark pathways were enriched in the high-creatinine group at FDR < 0.25, including 36 at FDR < 0.05, whereas no pathways were significantly enriched in the low-creatinine group. Prominent programs included TNFA signaling via NF-κB, interferon-γ and interferon-α responses, inflammatory response, IL6/JAK/STAT3 signaling, complement, hypoxia, apoptosis, reactive oxygen species, mTORC1 and PI3K/AKT/mTOR signaling, protein secretion, and the unfolded protein response (Figure 5e; Figure S4b; Table S15). Forty-one pathways retained FDR < 0.25 and concordant NES directions in both models, including 34 with FDR < 0.05 in both models. NES estimates were also concordant between models (Pearson r = 0.819; Spearman ρ = 0.872; Figure S4c).

ORA of the robust-core DEGs further supported this interpretation. Genes with higher expression in the high-serum-creatinine group were enriched for GO Biological Process terms related to autophagy, vacuole and vesicle organization, immune-response signaling, innate immune activation, and Golgi vesicle transport. KEGG analysis identified lysosomal, autophagic, and endocytic pathways, protein processing in the endoplasmic reticulum, TNF and NOD-like receptor signaling, and Fc gamma receptor-mediated phagosome formation. WikiPathways analysis additionally identified VEGFA–VEGFR2, EGF–EGFR, IL-4, TNF-α, oncostatin M, chemokine, and IL-1 signaling pathways (Supplementary Figure S4d–f; Supplementary Table S16). Disease- and infection-labeled pathways retained in the complete results were interpreted as shared host-response modules rather than evidence of specific pathogens or diseases.

#### 3.3.2. Continuous Modeling of Serum Creatinine Identifies a Robust Creatinine-Associated Urinary Transcriptomic Gradient

**Figure 6:**
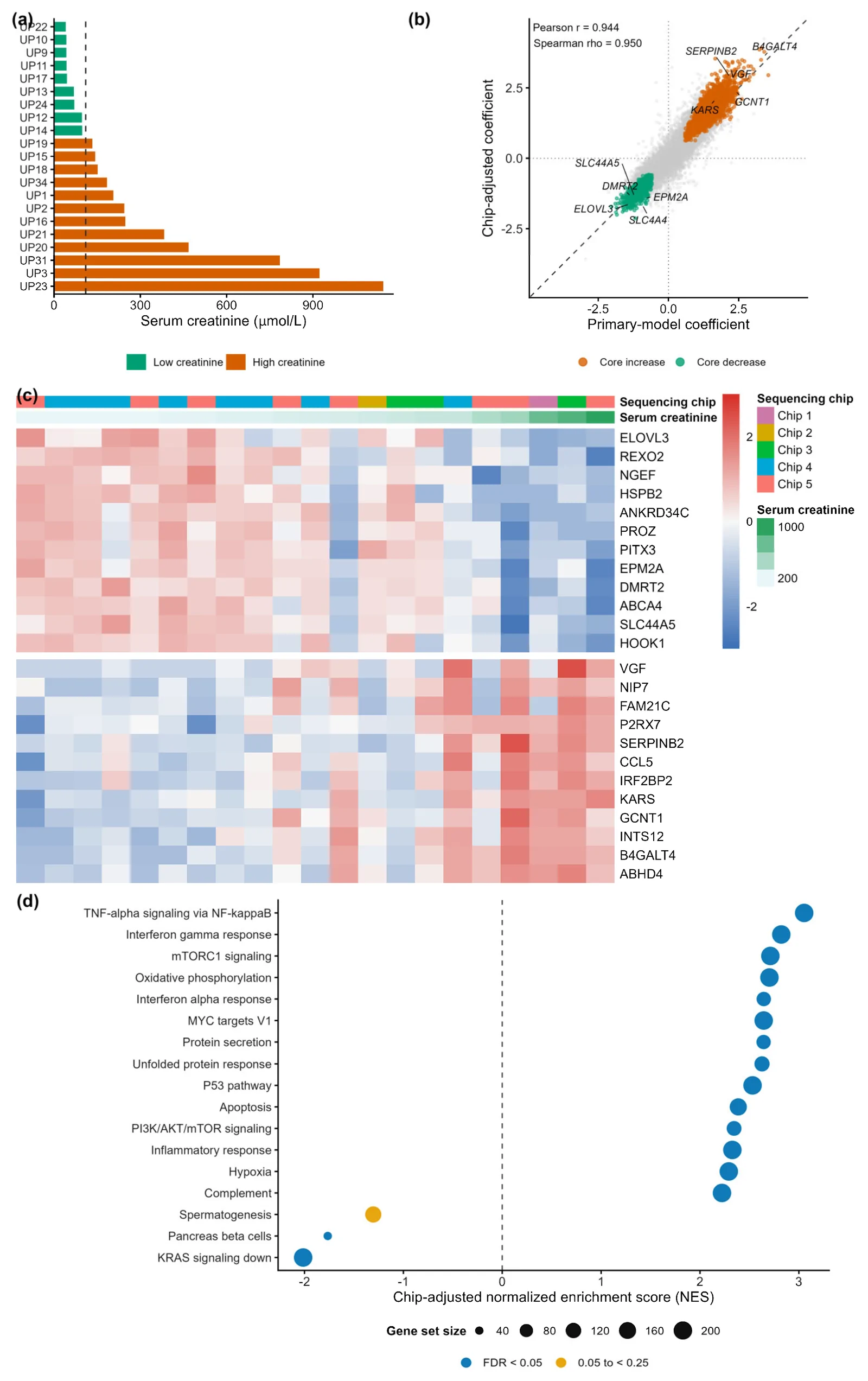
Continuous serum creatinine modeling identifies a robust creatinine-associated urinary transcriptomic gradient in sepsis. (**a**) Distribution of serum creatinine concentrations among the 21 patients with sepsis included in the urine RNA-seq analysis. Samples are ordered by increasing creatinine concentration. The dashed line indicates 110 µmol/L as a descriptive reference only; this threshold was not used in the continuous models. (**b**) Gene-level concordance between the primary and sequencing chip-adjusted continuous DESeq2 models. Each point represents a tested gene with finite coefficient estimates. Coefficients represent expression changes per one-standard-deviation increase in log2-transformed serum creatinine. Robust core genes met FDR < 0.05 and |coefficient| ≥ 0.58 in both models and had concordant directions. The dashed diagonal denotes equal coefficients (Pearson r = 0.944; Spearman ρ = 0.950). (**c**) Heatmap of 24 representative robust core genes, comprising 12 genes positively and 12 negatively associated with serum creatinine. Columns represent urine RNA-seq samples ordered by increasing creatinine concentration; rows represent genes clustered using row-scaled variance-stabilized expression values. Top annotations indicate serum creatinine concentration and sequencing chip. (**d**) Seventeen representative Hallmark pathways meeting FDR < 0.25 in both models and showing concordant NES directions. Point position indicates the NES from the chip-adjusted model, point size represents gene-set size, and color denotes the more conservative FDR category across the two models. Positive and negative NES values indicate enrichment among genes whose expression increased and decreased with serum creatinine, respectively. FDR, false discovery rate; GSEA, Gene Set Enrichment Analysis; NES, normalized enrichment score.

To determine whether the observed transcriptomic signal depended on dichotomization of serum creatinine, creatinine was modeled as a continuous variable in the 21 patients with sepsis for whom urine RNA-seq data were available. Creatinine concentrations were log2-transformed and standardized; consequently, the DESeq2 coefficient represented the log2 expression change associated with a one-standard-deviation increase in log2-transformed serum creatinine. Serum creatinine ranged from 40.4 to 1144 µmol/L, with a median of 143 µmol/L. For descriptive purposes, 12 patients had concentrations > 110 µmol/L and nine had concentrations ≤ 110 µmol/L (Figure 6a); this threshold was not used in the continuous models.

After expression prefiltering, 18,242 genes were tested. At an FDR < 0.05 and |coefficient| ≥ 0.58, the primary model identified 9053 creatinine-associated genes, including 5260 with positive and 3793 with negative coefficients. The sequencing chip-adjusted sensitivity model identified 4064 genes meeting the same criteria, including 2779 positively and 1285 negatively associated genes (Supplementary Figure S5a and Supplementary Table S17). A robust core set of 3774 genes met these criteria in both models and retained the same direction of association; 2570 showed increasing and 1204 showed decreasing expression with increasing serum creatinine.

Gene-level coefficients were highly concordant between the primary and chip-adjusted models across all tested genes with finite estimates (Pearson r = 0.944; Spearman ρ = 0.950; Figure 6b). Thus, although adjustment reduced the number of genes meeting the prespecified thresholds, the direction and magnitude of the estimated effects remained largely consistent, indicating that the creatinine-associated signal was not solely attributable to sequencing-chip structure.

Representative robust genes positively associated with serum creatinine included *B4GALT4*, *SERPINB2*, *GCNT1*, *MT1H*, *CCL3L3*, *IGJ*, *MT2A*, and *DDRGK1*. Conversely, *SLC44A5*, *SLC4A4*, *ELOVL3*, *EPM2A*, *HOOK1*, and *ABCA4* showed negative associations. These changes were consistent with increasing inflammatory and cellular-stress responses and decreasing transport, epithelial-signaling, and adhesion-related programs. Representative expression patterns are shown in Figure 6c and Supplementary Figure S5a–c, and complete gene-level results are provided in Supplementary Table S17.

Hallmark GSEA further demonstrated the pathway-level robustness of the continuous association. All 50 Hallmark gene sets were evaluated in both models. Forty-six pathways met an FDR < 0.25 in both models and had concordant NES directions, including 43 positively and three negatively enriched pathways; 39 also met the more stringent criterion of FDR < 0.05 in both models. Pathway-level NES values were highly correlated between models (Pearson r = 0.951; Spearman ρ = 0.969). Positive enrichment was observed for TNF-α signaling via NF-κB, interferon responses, inflammatory response, complement, hypoxia, apoptosis, unfolded protein response, oxidative phosphorylation, protein secretion, mTORC1 signaling, and PI3K–AKT–mTOR signaling. KRAS signaling down, pancreas beta cells, and spermatogenesis showed concordant negative enrichment (Figure 6d, Supplementary Figure S5d,e, and Supplementary Table S18).

ORA of the robust core genes positively associated with creatinine identified enrichment of macroautophagy, response to oxidative stress, vacuole and vesicle organization, autophagosome assembly, regulation of protein catabolism, and response to endoplasmic reticulum stress (Supplementary Figure S5f). KEGG enrichment included lysosome biogenesis, protein processing in the endoplasmic reticulum, TNF signaling, autophagy, mitophagy, phagocytosis, and endocytosis (Supplementary Figure S5g). WikiPathways enrichment included VEGFA–VEGFR2, IL-1, and macrophage-stimulating protein receptor signaling, regulation of the Warburg effect, SREBP signaling, and ferroptosis (Supplementary Figure S5h). Genes negatively associated with creatinine were predominantly enriched in sodium-ion transport, regulation of membrane potential, cell–cell adhesion, calcium and cadherin signaling, and proximal tubule transport. Complete ORA results are provided in Supplementary Table S19.

Together, the categorical and continuous analyses identified a consistent urinary transcriptomic axis associated with serum creatinine in sepsis. The categorical analysis demonstrated differences between the prespecified creatinine groups, whereas continuous modeling revealed a graded molecular association that remained highly concordant after sequencing-chip adjustment. Increasing creatinine was associated with inflammatory, interferon-related, autophagy–lysosomal, vesicle-trafficking, endoplasmic reticulum stress, and metabolic programs, whereas decreasing expression predominantly involved transport, adhesion, and epithelial-signaling modules.

#### 3.3.3. Sensitivity Analyses, Cross-Biofluid Comparison, and Prioritization of Transcripts for Orthogonal Validation

Exclusion of the sample with the highest serum creatinine concentration (1144 µmol/L) did not materially alter the continuous creatinine-associated transcriptional pattern. After exclusion (*n* = 20), 3192 of the 4064 genes identified in the original chip-adjusted analysis (78.5%) remained significant with the same direction of association, and 2926 of the 3774 original robust-core genes (77.5%) remained supported across the full and reduced analyses. Gene-level coefficients remained highly concordant with those from the complete cohort (Pearson r = 0.953; Spearman ρ = 0.945), as did Hallmark pathway NES values (Pearson r = 0.971; Spearman ρ = 0.973) (Supplementary Table S20).

Adjustment for demographic covariates also supported the stability of the creatinine-associated signal. Sex was available for all 21 patients and age for 20. The sex-adjusted, age-adjusted, and sex-plus-age-adjusted models identified 2872, 2358, and 2324 strict DEGs, respectively; 2324 genes retained a consistent effect direction across all evaluated models. Exploratory sex-by-creatinine interaction analysis identified only three genes at FDR < 0.05 (*AIFM1*, *ATM*, and *TYRO3*) (Supplementary Table S21).

To determine whether the urinary creatinine-associated transcriptional pattern was mirrored in whole blood, continuous creatinine effects were compared in matched blood and urine samples from the same 21 patients. After adjustment for sequencing batch or chip, respectively, 31 strict DEGs were identified in blood compared with 4064 in urine. Among 12,492 genes tested in both biofluids, adjusted gene-level coefficients showed weak concordance (Pearson r = 0.192; Spearman ρ = 0.199), with similarly weak concordance of Wald statistics (Pearson r = 0.190; Spearman ρ = 0.211). Only five strict DEGs overlapped between the two biofluids; two showed concordant and three discordant directions of association. None of the 10 blood robust-core genes overlapped with the 3774-gene urinary robust core. At the pathway level, cross-biofluid concordance was greater but remained moderate (Hallmark NES Pearson r = 0.293; Spearman ρ = 0.464). Fourteen of the 15 robust Hallmark pathways identified in blood were also robust in urine, whereas 46 robust pathways were identified in urine overall (Supplementary Figure S6 and Supplementary Table S23).

Applying the predefined statistical and technical prioritization criteria yielded 1267 technically favorable candidates. The final panel comprised five transcripts positively associated with serum creatinine (*SERPINB2*, *IRF2BP2, CSF1*, *P2RX7*, and *FAM46C*) and five negatively associated transcripts (*SLC44A5, SLC4A4*, *NGEF*, *EPM2A*, and *ABCA4*). None showed a significant sex-by-creatinine interaction, and no manual substitutions were made. These transcripts were prioritized for subsequent orthogonal validation and were not considered validated biomarkers (Supplementary Table S22). In the matched blood analysis, seven of these 10 transcripts passed the blood expression prefilter, but none belonged to the blood robust core; *SLC44A5*, *NGEF*, and *ABCA4* were not tested in blood after expression prefiltering (Supplementary Table S23).

## 4. Discussion

This comparative targeted RNA-seq study identified distinct but partially overlapping host-response patterns in whole blood and the urinary cellular fraction. The whole-blood transcriptomic profile primarily reflected systemic immune dysregulation, characterized by myeloid and neutrophil activation and suppression of adaptive immune programs. The transcriptomic profile of the urinary cellular fraction showed inflammatory, epithelial- and barrier-related, transport, and metabolic expression patterns.

Although the two biofluids shared a concordant inflammatory and myeloid core, most transcriptomic changes were biofluid-specific. Of 336 shared DEGs, 314 (93.5%) changed in the same direction, whereas 1460 and 1216 DEGs were specific to blood and urine, respectively. Paired analysis identified biofluid-associated differences in 15,162 of 20,580 tested genes, demonstrating extensive biofluid-associated expression differences within individuals. However, these differences may substantially reflect the intrinsic cellular composition and biological origin of blood and urinary cellular fractions and should not be interpreted as disease-specific compartmentalization.

A major finding was a robust urinary transcriptional axis associated with serum creatinine. Both threshold-based and continuous models demonstrated extensive transcriptomic remodeling that persisted after adjustment for sequencing chip. Increasing creatinine was associated with inflammatory, interferon, hypoxic, oxidative-stress, autophagic and lysosomal, vesicle-trafficking, endoplasmic reticulum stress, and metabolic programs, whereas decreasing expression involved tubular transport, ion homeostasis, epithelial signaling, and cell–cell adhesion. These findings support a layered multi-biofluid model in which whole blood reflects systemic immune status, whereas the urinary cellular-fraction profile shows distinct creatinine-associated, inflammatory, epithelial/barrier-related, transport, and metabolic patterns.

### 4.1. Blood Transcriptome Reflects Systemic Myeloid Activation and Adaptive Immune Suppression

The blood transcriptome showed pronounced myeloid and neutrophil activation accompanied by suppression of adaptive immune and T-cell-associated programs. Increased expression of *CD177*, *IL1R2*, *MMP9*, *S100A12*, *ANXA3*, *HK3*, *VNN1*, and *IRAK3* was associated with phagocytosis, cytokine production, responses to bacterial components, TNF-α/NF-κB and IL-6/JAK/STAT3 signaling, complement, coagulation, and hypoxia. Conversely, downregulated genes were enriched in lymphocyte activation, T-cell differentiation, antigen receptor signaling, and ribosomal and translational processes. Persistence of 1796 robust core DEGs after adjustment for sequencing chip supports the biological stability of this profile.

This myeloid-dominant signature is consistent with bulk and single-cell studies implicating neutrophils and monocytes in sepsis pathogenesis. Adverse outcomes have been associated with necrotic and pyroptotic programs in neutrophils and with apoptotic, autophagic, and ferroptotic programs in monocytes [48]. Whole-blood studies have also linked mortality to neutrophil degranulation independently of absolute neutrophil counts, suggesting altered cellular function rather than abundance alone [49]. Single-cell analyses have identified prognostically relevant remodeling of dendritic-cell, plasmablast, monocyte, and lymphoid compartments, including enhanced TNF-α/NF-κB/AP-1 activity and impaired interferon regulation [50].

Suppression of adaptive immunity is similarly consistent with previous observations. Recovery from sepsis has been associated with restoration of immune pathways and T-cell receptor diversity [51], whereas persistent lymphoid immunosuppression, chronic inflammation, metabolic dysfunction, and impaired T-cell signaling characterize late or complicated disease [52,53]. Cell-specific RNA-seq has further demonstrated suppression of immune-response pathways, increased expression of immunosuppressive mediators such as *ARG1*, *SOCS1*, and *SOCS3*, and upregulation of inhibitory checkpoints including *TIGIT, LAG3, PDCD1*, and *CTLA4* [54].

The resulting myeloid-high, adaptive-low profile is compatible with established MARS and consensus transcriptomic subtypes, which differ in inflammatory, neutrophil, heme and coagulation, interferon, and lymphocyte programs [10,26]. Because formal subtype assignment was not performed, these findings indicate biological concordance with existing endotyping frameworks rather than membership in a specific subtype.

### 4.2. Transcriptomic Profiling of the Urinary Cellular Fraction Reveals Inflammatory and Epithelial- or Barrier-Related Programs

The transcriptomic profile of the urinary cellular fraction combined inflammatory and myeloid activation with epithelial, barrier-related, and metabolic alterations. Upregulated genes included *HP*, *IL18RAP*, *TLR5*, *HK3*, *FCGR1A*, *ARG1*, *PTX3*, and *CXCL5* and were enriched in myeloid leukocyte activation, migration, chemotaxis, phagocytosis, IL-1β-associated signaling, and responses to bacteria. These findings indicate a substantial inflammatory and myeloid component in the urinary cellular fraction.

Downregulated programs involved epithelial differentiation, tight junctions, cadherin signaling, transport, and metabolism. Keratinocyte- or epidermal-like enrichment terms probably represent shared epithelial and barrier transcriptional programs rather than skin-specific signals and are compatible with urothelial or other epithelial cells in urinary sediment.

These findings agree with urinary cellular transcriptomic studies reporting an early 239-gene sepsis signature involving innate immunity, cell migration, hypoxia, oxidative stress, and markers of myeloid and tubular epithelial origin [35]. Urinary expression profiles have also distinguished sepsis from sterile systemic inflammation and revealed reduced oxidative phosphorylation, amino-acid metabolism, and lipid and fatty-acid oxidation [36]. Urine may therefore capture epithelial and metabolic remodeling in addition to inflammation.

Interpretation of the urine sepsis-versus-control comparison nevertheless requires caution because sequencing chip and clinical condition were substantially confounded, precluding reliable separation of biological and technical effects. The quantitative magnitude and exact gene composition of this exploratory signature therefore require independent replication. Nevertheless, its functional patterns are biologically plausible and consistent with previously reported inflammatory and epithelial-related urinary responses in sepsis.

### 4.3. Blood and Urine Share an Inflammatory Core but Show Extensive Biofluid-Specific Expression Differences

Comparison of 1796 robust blood DEGs with 1552 urinary DEGs identified 336 shared genes, representing 18.7% and 21.6% of the respective signatures. Of these, 314 (93.5%) were concordant: 254 were upregulated and 60 downregulated in both biofluids, whereas only 22 showed opposing effects. Concordantly upregulated genes, including *IL18RAP*, *TLR5*, *HK3*, *FCGR1A*, *CST7*, *ALPL*, *MS4A6A*, *NFE2*, *FKBP5*, and *C19orf59*, defined a shared inflammatory and myeloid core involving innate immune activation, IL-18 and Toll-like receptor signaling, Fc-receptor signaling, phagocytosis, cytokine production, and inflammatory metabolic remodeling.

Most alterations nevertheless remained biofluid-specific. Blood-specific downregulated genes were associated with adaptive immunity, T-cell activation, antigen receptor signaling, and lymphocyte differentiation. Urine-specific upregulated genes involved leukocyte migration, chemotaxis, cytokine signaling, responses to lipopolysaccharide, and antimicrobial defense, whereas urine-specific downregulated genes reflected epithelial, barrier-related, and metabolic functions.

Paired analysis of 21 patients confirmed extensive expression differences between blood and urine. After accounting for patient identity, 15,162 of 20,580 genes (73.7%) differed between the two biofluids; 7740 were more highly expressed in blood and 7422 in urine. Blood-enriched genes involved RNA processing, translation, immunity, and platelet or megakaryocyte functions, whereas urine-enriched genes involved receptor and G-protein-coupled receptor signaling, epithelial- and barrier-related processes, ion transport, and antimicrobial responses. Persistence of these differences within individuals excludes interpatient heterogeneity as their sole explanation, but does not distinguish constitutive differences in biofluid cellular composition from sepsis-related effects.

The shared signature is supported by previous identification of *TLR5* and *FCGR1A* as myeloid or macrophage-associated hub genes in sepsis [55] and by expansion of the MS1 CD14-positive monocyte state in single-cell studies [56]. Dynamic changes in MS1-like monocytes, developing neutrophils, and lymphopenia-associated programs have also been linked to recovery and mortality [57]. Blood-specific expression of platelet and megakaryocyte genes, including *PPBP*, *PF4*, *TUBB1*, and *ITGB3*, is consistent with extensive platelet transcriptomic and translational remodeling in sepsis [58]. Together, these observations support a shared inflammatory response superimposed on extensive biofluid-specific expression structure.

### 4.4. A Creatinine-Associated Urinary Transcriptomic Axis Shows a Continuous Molecular Gradient

The strongest clinical–transcriptomic association involved serum creatinine. Threshold-based analysis at 110 µmol/L identified 4990 robust-core strict DEGs that retained significance and direction after adjustment for sequencing chip. Of these, 4777 showed higher expression and 213 showed lower expression in the high-creatinine group. Genes with higher expression were associated with inflammatory, innate immune, hypoxic, apoptotic, autophagic and lysosomal, vesicle-trafficking, and cellular stress-response programs.

Continuous modeling demonstrated that this signature represented a directional molecular gradient rather than only a contrast between predefined groups. After chip adjustment, 4064 DEGs remained significant, including 3774 core strict DEGs with strongly concordant effects between the primary and adjusted models. Increasing creatinine was associated with TNF-α/NF-κB signaling, interferon responses, inflammation, complement, hypoxia, apoptosis, oxidative phosphorylation, the unfolded protein response, mTORC1 signaling, autophagy, lysosome biogenesis, mitophagy, endocytosis, and ferroptosis. Decreasing expression involved proximal tubular and sodium transport, membrane-potential regulation, calcium and cadherin signaling, and epithelial adhesion.

The continuous association was not driven by the sample with the highest serum creatinine concentration. After exclusion of this sample (1144 µmol/L), 3192 of 4064 genes (78.5%) from the original chip-adjusted analysis remained significant with the same direction of association, and 2926 of 3774 original robust-core genes (77.5%) remained supported across the full and reduced analyses. Gene-level coefficients remained highly concordant (Pearson r = 0.953; Spearman ρ = 0.945), as did Hallmark pathway NES values (Pearson r = 0.971; Spearman ρ = 0.973).

Adjustment for available demographic covariates provided additional support for robustness. Sex-, age-, and sex-plus-age-adjusted models identified 2872, 2358, and 2324 strict DEGs, respectively, with 2324 genes retaining a consistent direction across all evaluated models. Only three genes showed a significant sex-by-creatinine interaction at FDR < 0.05, and none belonged to the final prioritized transcript panel.

The simultaneous enrichment of inflammatory and stress-response pathways together with reduced representation of transport-related programs is compatible with coordinated molecular variation along the serum creatinine gradient. However, serum creatinine was not accompanied by formal AKI adjudication or cellular-resolution measurements of the urinary sediment. Accordingly, the identified axis should be interpreted as a serum creatinine-associated urinary transcriptional gradient rather than as a kidney-specific injury signature or a validated diagnostic or prognostic classifier of sepsis-associated AKI.

The matched blood–urine analysis provided additional context for interpreting this axis. Despite evaluation of the same 21 patients and the same serum creatinine measurements, adjusted gene-level effects showed only weak cross-biofluid concordance, with five overlapping strict DEGs and no overlap between the blood and urine robust cores. In contrast, 14 of the 15 robust Hallmark pathways identified in blood were also robust in urine, although urine showed a substantially broader set of robust enriched pathways overall. This combination of marked gene-level divergence and partial pathway-level convergence suggests that related higher-order biological processes may be represented through substantially different transcriptional responses in the two biofluids. Importantly, these findings indicate that the urinary creatinine-associated axis is not strongly mirrored by the whole-blood transcriptional response, but they do not establish kidney specificity because the cellular composition and tissue origin of the urinary signal remain unresolved.

Independent studies support the biological plausibility of urine-based molecular profiling. Urinary extracellular-vesicle proteomics identified CD35-positive vesicles as a potential marker of sepsis-associated AKI, severity, renal recovery, and complement-associated podocyte injury [59]. Urinary single-cell RNA-seq has shown that shed tubular epithelial cells retain transcriptional features of oxidative stress, inflammation, injury, and repair [32]. Renal tissue and experimental models further demonstrate mitochondrial dysfunction, metabolic remodeling, immune-cell infiltration, and compartment-specific inflammatory and coagulation responses [60,61]. Single-cell and spatial transcriptomics during endotoxemia have also shown a shift of proximal tubular cells from solute transport and homeostasis toward immune and antigen-presentation programs [62]. These observations provide mechanistic context but do not establish the cellular origin of the present bulk urinary signature.

Blood transcriptomic profiles have previously helped distinguish transient from persistent acute kidney injury and improved trajectory prediction beyond clinical variables alone [63]. The present findings extend this concept to RNA derived from the urinary cellular fraction by identifying a continuous transcriptional signal integrating inflammation, cellular stress, autophagic and lysosomal activity, metabolic remodeling, and reduced transport-related expression.

### 4.5. Strengths and Limitations

The principal strengths of this study include prospective recruitment at three hospitals, integrated profiling of blood and urinary cellular fractions, and the availability of paired blood–urine samples from patients with sepsis. The analytical strategy combined primary and technically adjusted models, categorical and continuous creatinine analyses, gene-level differential expression, pathway enrichment, and explicit assessment of cross-model robustness. Additional sensitivity analyses addressed the influence of the highest serum creatinine value and potential confounding by sex and age. The version-controlled workflow and documented software environment further improve analytical transparency and reproducibility. The matched cross-biofluid continuous-creatinine analysis further allowed the urinary association to be evaluated against the corresponding whole-blood transcriptional response within the same patients.

Several limitations should be considered. First, the cohort was relatively small, particularly for the urinary and paired analyses, and no independent validation cohort was available. The creatinine-associated analyses should therefore be regarded as exploratory. Second, the targeted RNA-seq panel did not provide complete transcriptome coverage and may have limited the detection of unrepresented genes and pathways.

Third, sequencing chip and clinical condition were substantially confounded in the urine sepsis-versus-control comparison, precluding an interpretable batch-adjusted model for that contrast. Although the creatinine-associated findings remained robust after chip adjustment and additional sensitivity analyses, the urine case–control signature requires independent replication. Fourth, the control group was smaller and differed from the sepsis group in both age and sex distribution. Age- and sex-related transcriptional differences may therefore have contributed to the unadjusted case–control contrasts.

Fifth, RNA-seq was performed on bulk whole blood and the urinary cellular fraction. Differences in leukocyte, renal epithelial, urothelial, and other cell populations could contribute to the observed signatures, and their cellular origins cannot be resolved without deconvolution or single-cell measurements. Urine sediment cellular composition and routine urine microscopy data, including hematuria and pyuria, were not available for the RNA-seq cohort. The paired analysis also cannot fully distinguish constitutive differences in biofluid cellular composition from disease-induced biofluid-specific responses.

Because blood and urinary cellular RNA differ in expression detectability, dispersion structure, cellular composition, and technical characteristics, the difference in the absolute numbers of creatinine-associated DEGs between the two biofluids should not be interpreted as a direct quantitative measure of relative biological effect magnitude.

Finally, samples were collected at a single clinical time point. Formal KDIGO AKI classification and serial urine-output data were not available for the urinary RNA-seq cohort, precluding definitive AKI adjudication. Complete SOFA scores and vasopressor-exposure data were also unavailable, limiting adjustment for overall illness severity. Consequently, the creatinine-associated transcriptional axis cannot be fully separated from other clinical correlates of disease severity. The observational design further precludes causal interpretation or assessment of diagnostic and prognostic performance.

### 4.6. Clinical Implications and Future Directions

The results support a multi-layered view of sepsis in which whole-blood profiles predominantly reflect systemic myeloid activation and adaptive immune suppression, whereas transcriptomic profiles of the urinary cellular fraction show distinct inflammatory, epithelial- and barrier-related, transport, and metabolic patterns. Combined profiling may therefore characterize sepsis heterogeneity more comprehensively than either biofluid alone, although biofluid-specific differences must be interpreted in the context of their distinct cellular composition and biological origin. The weak gene-level concordance of creatinine-associated effects between matched blood and urine further suggests that the transcriptomic profile of the urinary cellular fraction contains molecular information not strongly represented in the corresponding whole-blood profile, although the biological source of this information remains unresolved.

The creatinine-associated urinary gradient is of potential clinical interest because it links a routinely available clinical measure with coordinated inflammatory, stress-response, metabolic, epithelial/barrier-related, and transport-associated transcriptional variation. These patterns are not kidney-specific and are not yet diagnostic or prognostic tests; they should complement, rather than replace, clinical assessment, microbiological diagnostics, organ-function monitoring, and standard sepsis management [3].

Further studies should validate these findings in larger prospective multicenter cohorts with serial blood and urine sampling. Baseline renal function, creatinine kinetics, urine output, AKI stage, treatment, and clinical outcomes should be evaluated concurrently to establish temporal and prognostic associations. The 10 prioritized transcripts identified here provide a focused candidate set for orthogonal validation by RT-qPCR or digital PCR, but should not be regarded as validated biomarkers at this stage. Computational deconvolution, flow cytometry, and single-cell RNA-seq could help resolve the cellular sources of urinary signals. Integration with proteomics, metabolomics, and extracellular-vesicle profiling may ultimately enable molecular stratification combining systemic host responses with urinary molecular phenotypes and clinically measured organ function [64].

## 5. Conclusions

Transcriptomic profiles of whole blood and urinary cellular fractions showed distinct but partially overlapping host-response patterns in sepsis. Whole blood predominantly reflected systemic myeloid activation and adaptive immune suppression, whereas the urinary cellular-fraction profile showed inflammatory, epithelial- and barrier-related, transport, and metabolic expression patterns. Although the two biofluids shared inflammatory programs, their extensive expression differences should be interpreted in the context of their distinct cellular composition and biological origin.

A robust urinary transcriptional gradient was associated with serum creatinine and persisted after sequencing-chip adjustment, exclusion of the highest creatinine value, and adjustment for sex and age. In matched blood and urine from the same 21 patients, creatinine-associated gene-level effects showed weak cross-biofluid concordance, with only five overlapping strict DEGs and no overlap between robust cores, whereas pathway-level concordance was greater but remained moderate. These findings suggest that the urinary creatinine-associated transcriptional axis is not strongly mirrored by the corresponding whole-blood response, while not establishing kidney specificity. Because complete clinical severity covariates were unavailable, the creatinine-associated transcriptional axis cannot be fully separated from other clinical correlates of disease severity. The identified patterns remain exploratory and are not validated diagnostic or prognostic signatures. Independent longitudinal cohorts with detailed renal phenotyping and orthogonal validation of the prioritized transcripts are required to determine their biological and clinical relevance.

## Figures and Tables

**Table 1:** Clinical characteristics of the final RNA-seq cohort and sample availability.

Characteristic	Sepsis (*n* = 35)	Controls (*n* = 10)
**Demographic** **characteristics**		
Age, years, median [IQR] (range)	63.5 [51.3–69.8] (25–88)	44.5 [39.8–56.8] (25–66)
Male sex, *n*/*N* (%)	24/35 (68.6%)	2/10 (20.0%)
**RNA-seq sample availability**		
Participants with blood RNA-seq, *n*	35	10
Participants with urine RNA-seq, *n*	21	7
Participants with paired blood–urine RNA-seq profiles, *n*	21	7
**Clinical characteristics**		
In-hospital mortality, *n*/*N* (%)	18/35 (51.4%)	-
Mechanical ventilation, *n*/*N* (%)	18/35 (51.4%)	-
Positive microbiological culture, *n*/*N* (%)	13/35 (37.1%)	-
Serum creatinine, µmol/L, median [IQR] (range)	133.0 [69.7–225.3] (40.4–1144.0)	**-**
Serum creatinine group, >110/≤110 µmol/L, *n*	20/15	**-**

Data are presented as *n*/*N* (%) or median [interquartile range] (range), as appropriate. Serum creatinine summaries in Table 1 refer to all 35 patients with sepsis. Creatinine concentrations > 110 µmol/L and ≤110 µmol/L were classified as high and low, respectively. The creatinine-associated urinary transcriptomic analyses were restricted to the 21 patients with available urinary RNA-seq profiles. RNA-seq, RNA sequencing.

## Data Availability

De-identified gene-level expression matrices and non-sensitive sample metadata generated in this study have been deposited in the NCBI Gene Expression Omnibus (GEO) under accession GSE345856, with the corresponding raw sequencing data deposited in the NCBI Sequence Read Archive (SRA). These records are currently available to reviewers through private-access mechanisms and will be released publicly in accordance with NCBI policies. Analysis code is available at https://github.com/beloslav1976-svg/targeted-sepsis-transcriptomics, accessed on 6 October 2026, with the manuscript-associated release v1.1.0 archived in Zenodo at https://doi.org/10.5281/zenodo.22096555. Additional individual-level clinical data are not publicly available because the informed-consent documentation did not authorize unrestricted sharing and may be made available subject to institutional and ethics approval.

## Supplementary Materials

The following supporting information can be downloaded at https://www.mdpi.com/article/10.3390/diagnostics16203410/s1. Table S1: Detailed de-identified annotation of study samples. Table S2: Complete DESeq2 results and model concordance for the blood sepsis-versus-control comparison; Table S3: Robust blood core differentially expressed genes and representative sepsis-associated genes; Table S4: Assessment of sex chromosome-linked and Y-linked outlier genes in the blood sepsis-versus-control analysis; Table S5: GO, KEGG, and WikiPathways enrichment results for robust blood core differentially expressed genes; Table S6: Hallmark GSEA results for the blood sepsis-versus-control comparison; Figure S1: Blood sepsis-versus-control transcriptomic analysis: (a) principal component analysis of blood samples according to sequencing chip, with disease condition indicated by point shape; (b) MA plot for the primary DESeq2 model; (c) DESeq2 dispersion estimates; (d) sample-to-sample distance heatmap; (e) concordance of gene-level log2 fold-change estimates between the primary and chip-adjusted DESeq2 models; (f) GO Biological Process enrichment; (g) KEGG pathway enrichment; and (h) WikiPathways enrichment of robust blood core differentially expressed genes; Table S7: Differential expression results for urine sepsis versus control; Table S8: Functional enrichment of urine sepsis-associated differentially expressed genes; Table S9: Hallmark GSEA of urine sepsis versus control; Figure S2: Exploratory urine sepsis-versus-control transcriptomic analysis: (a) principal component analysis of urine samples according to sequencing chip; (b) MA plot; (c) DESeq2 dispersion estimates; (d) GO Biological Process over-representation analysis; (e) KEGG pathway over-representation analysis; and (f) WikiPathways over-representation analysis of urine sepsis-associated differentially expressed genes; Table S10: Cross-biofluid comparison of blood and urine sepsis-associated differential expression signatures; Table S11: GO Biological Process enrichment of shared and biofluid-specific sepsis-associated gene modules; Table S12: Paired blood-versus-urine differential expression analysis in patients with sepsis; Table S13: Functional enrichment of blood-enriched and urine-enriched genes in the paired analysis; Figure S3: Shared and biofluid-specific transcriptomic programs: (a) representative concordantly regulated sepsis-associated genes shared between blood and urine, showing blood and urine log2 fold-change estimates; (b) principal component analysis of matched blood and urine RNA-seq profiles; and (c) volcano plot of the paired blood-versus-urine differential expression analysis; Table S14: Differential expression results for serum creatinine-stratified urine samples; Table S15: Hallmark GSEA of serum creatinine-stratified urine samples; Table S16: Functional enrichment of creatinine-associated core urine differentially expressed genes; Figure S4: Categorical serum creatinine-associated urinary transcriptomic analysis: (a) principal component analysis according to sequencing chip and serum creatinine group; (b) expanded chip-adjusted Hallmark GSEA; (c) concordance of Hallmark normalized enrichment scores between the primary and chip-adjusted models; and over-representation analysis of robust-core urine genes with higher expression in the high-creatinine group for (d) GO Biological Process, (e) KEGG, and (f) WikiPathways; Table S17: Continuous serum creatinine-associated differential expression results in urine; Table S18: Hallmark GSEA results for the continuous serum creatinine model; Table S19: GO Biological Process, KEGG, and WikiPathways over-representation analysis of continuous serum creatinine-associated urine genes; Figure S5: Continuous serum creatinine-associated urinary transcriptomic analysis: (a) volcano plot of genes associated with continuous serum creatinine in the chip-adjusted model; (b) expression trends of representative creatinine-associated genes; (c) classification of genes according to their support across the primary and chip-adjusted models; (d) expanded Hallmark GSEA; (e) concordance of Hallmark normalized enrichment scores between the continuous creatinine models; and over-representation analysis of continuous serum creatinine-associated urine genes for (f) GO Biological Process, (g) KEGG, and (h) WikiPathways; Table S20: Sensitivity analysis of the continuous serum creatinine-associated urinary transcriptomic signal after exclusion of the sample with the highest serum creatinine concentration; Table S21: Demographic covariate sensitivity and sex-by-creatinine interaction analyses; Table S22: Prioritization of serum creatinine-associated urinary transcripts for subsequent orthogonal validation; Table S23: Matched blood–urine comparison of continuous serum creatinine-associated transcriptomic effects, including gene-level concordance, shared strict DEGs, Hallmark pathway comparison, and cross-biofluid evaluation of the prioritized transcript panel; Figure S6: Cross-biofluid comparison of serum creatinine-associated transcriptomic effects in matched blood and urine samples: (a) comparison of sequencing-adjusted gene-level effects in blood and urine, including strict-DEG overlap and directionality; and (b) comparison of Hallmark normalized enrichment scores and robust pathway membership between blood and urine.

## References

[1] Modugula S., Altenbaugh M., Ivanova M., DuMont T., Arshad H. (2025). Sepsis Epidemiology, Definitions, Scoring Systems, and Diagnostic Markers. Crit. Care Nurs. Q..

[2] Singer M., Angus D.C., Annane D., Bauer M., Kalil A.C., Klompas M., Machado F.R., Martin G.S., Randolph A.G., Shankar-Hari M. (2026). Sepsis. Lancet.

[3] Prescott H.C., Antonelli M., Alhazzani W., Møller M.H., Alshamsi F., Azevedo L.C.P., Belley-Cote E., De Waele J., Derde L., Dionne J.C. (2026). Surviving Sepsis Campaign: International Guidelines for Management of Sepsis and Septic Shock 2026. Crit. Care Med..

[4] Silva M.J., Fehrenbach G.W., Pogue R., Murray P., Rezoagli E., Laffey J.G., Murphy E.J. (2026). Sepsis Modelling: Current Approaches and Organ-on-Chip Perspectives. eBioMedicine.

[5] Gray A.P., Chung E., Hsu R.L., Araki D.T., Gershberg Hayoon A., Davis Weaver N., Swetschinski L.R., Wool E.E., Han C., Mestrovic T. (2025). Global, Regional, and National Sepsis Incidence and Mortality, 1990–2021: A Systematic Analysis. Lancet Glob. Health.

[6] Saito H. (2025). Sepsis in Global Health: Current Global Strategies to Fight against Sepsis. Acute Med. Surg..

[7] Kolesnichenko S.I., Lavrinenko A.V., Akhmaltdinova L.L. (2021). Bloodstream Infection Etiology among Children and Adults. Int. J. Microbiol..

[8] Stanski N.L., Wong H.R. (2020). Prognostic and Predictive Enrichment in Sepsis. Nat. Rev. Nephrol..

[9] Stevens R.D., Harris C. (2025). Unlocking Host Response Signatures in Sepsis and Critical Illnesses. Nat. Med..

[10] Scicluna B.P., Cano-Gamez K., Burnham K.L., Davenport E.E., Moore A.R., Khan S., Hinds C.J., Cremer O.L., Khatri P., Sweeney T.E. (2025). A Consensus Blood Transcriptomic Framework for Sepsis. Nat. Med..

[11] Cavaillon J., Singer M., Skirecki T. (2020). Sepsis Therapies: Learning from 30 Years of Failure of Translational Research to Propose New Leads. EMBO Mol. Med..

[12] Moore A.R., Zheng H., Ganesan A., Hasin-Brumshtein Y., Maddali M.V., Levitt J.E., Van Der Poll T., Lu J., Bouma H.R., Scicluna B.P. (2025). A Consensus Immune Dysregulation Framework for Sepsis and Critical Illnesses. Nat. Med..

[13] Marshall J.C. (2014). Why Have Clinical Trials in Sepsis Failed?. Trends Mol. Med..

[14] McCurdy M.T., Sweeney T.E., Foster D., Reddy B., Simpson S.Q., for Sepsis Alliance and the Infection Management and Sepsis Collaborative Community (IMSCC) Panelists (2025). Challenges Facing Developers of Diagnostic Tests for Sepsis: A Report From Sepsis Alliance and the Infection Management and Sepsis Collaborative Community. Crit. Care Explor..

[15] Duncan C.F., Youngstein T., Kirrane M.D., Lonsdale D.O. (2021). Diagnostic Challenges in Sepsis. Curr. Infect. Dis. Rep..

[16] Sweeney T.E., Perumal T.M., Henao R., Nichols M., Howrylak J.A., Choi A.M., Bermejo-Martin J.F., Almansa R., Tamayo E., Davenport E.E. (2018). A Community Approach to Mortality Prediction in Sepsis via Gene Expression Analysis. Nat. Commun..

[17] Liesenfeld O., Arora S., Aufderheide T.P., Clements C.M., DeVos E., Fischer M., Giamarellos-Bourboulis E.J., House S., Humphries R.L., Gill J.K. (2025). Clinical Validation of an AI-Based Blood Testing Device for Diagnosis and Prognosis of Acute Infection and Sepsis. Nat. Med..

[18] Davenport E.E., Burnham K.L., Radhakrishnan J., Humburg P., Hutton P., Mills T.C., Rautanen A., Gordon A.C., Garrard C., Hill A.V.S. (2016). Genomic Landscape of the Individual Host Response and Outcomes in Sepsis: A Prospective Cohort Study. Lancet Respir. Med..

[19] Hancock R.E.W., An A., Dos Santos C.C., Lee A.H.Y. (2025). Deciphering Sepsis: Transforming Diagnosis and Treatment through Systems Immunology. Front. Sci..

[20] Rashid A., Al-Obeidat F., Kanthimathinathan H.K., Benakatti G., Hafez W., Ramaiah R., Brierley J., Hanisch B., Khilnani P., Koutentis C. (2024). Advancing Sepsis Clinical Research: Harnessing Transcriptomics for an Omics-Based Strategy—A Comprehensive Scoping Review. Inform. Med. Unlocked.

[21] Tidswell R., Inada-Kim M., Singer M. (2021). Sepsis: The Importance of an Accurate Final Diagnosis. Lancet Respir. Med..

[22] Cano-Gamez E., Burnham K.L., Goh C., Allcock A., Malick Z.H., Overend L., Kwok A., Smith D.A., Peters-Sengers H., Antcliffe D. (2022). An Immune Dysfunction Score for Stratification of Patients with Acute Infection Based on Whole-Blood Gene Expression. Sci. Transl. Med..

[23] Sweeney T.E., Azad T.D., Donato M., Haynes W.A., Perumal T.M., Henao R., Bermejo-Martin J.F., Almansa R., Tamayo E., Howrylak J.A. (2018). Unsupervised Analysis of Transcriptomics in Bacterial Sepsis Across Multiple Datasets Reveals Three Robust Clusters. Crit. Care Med..

[24] Wong H.R., Cvijanovich N.Z., Anas N., Allen G.L., Thomas N.J., Bigham M.T., Weiss S.L., Fitzgerald J., Checchia P.A., Meyer K. (2015). Developing a Clinically Feasible Personalized Medicine Approach to Pediatric Septic Shock. Am. J. Respir. Crit. Care Med..

[25] Yao L., Rey D.A., Bulgarelli L., Kast R., Osborn J., Van Ark E., Fang L.T., Lau B., Lam H., Teixeira L.M. (2022). Gene Expression Scoring of Immune Activity Levels for Precision Use of Hydrocortisone in Vasodilatory Shock. Shock.

[26] Scicluna B.P., Van Vught L.A., Zwinderman A.H., Wiewel M.A., Davenport E.E., Burnham K.L., Nürnberg P., Schultz M.J., Horn J., Cremer O.L. (2017). Classification of Patients with Sepsis According to Blood Genomic Endotype: A Prospective Cohort Study. Lancet Respir. Med..

[27] Burnham K.L., Davenport E.E., Radhakrishnan J., Humburg P., Gordon A.C., Hutton P., Svoren-Jabalera E., Garrard C., Hill A.V.S., Hinds C.J. (2017). Shared and Distinct Aspects of the Sepsis Transcriptomic Response to Fecal Peritonitis and Pneumonia. Am. J. Respir. Crit. Care Med..

[28] Cheng Y., Xu L., Wang J., Cao X., Chen D., Zhang P., Yang L., Qin L. (2023). Analysis of Bulk RNA-Seq Data from Sepsis Patients Reveals Sepsis-Associated lncRNAs and Targeted Cell Death-Related Genes Contributing to Immune Microenvironment Regulation. Front. Immunol..

[29] Fratea A., Riza A.-L., Dumitrescu F., Dorobantu S., Pirvu A., Dragos A., Grigorescu A., Streata I., Netea M.G., Kumar V. (2026). Integrated Transcriptomic and Proteomic Profiling Identifies an Interferon-Dependent Inflammatory Endotype in Sepsis. Biomed. Pharmacother..

[30] Antcliffe D.B., Burnham K.L., Al-Beidh F., Santhakumaran S., Brett S.J., Hinds C.J., Ashby D., Knight J.C., Gordon A.C. (2019). Transcriptomic Signatures in Sepsis and a Differential Response to Steroids. From the VANISH Randomized Trial. Am. J. Respir. Crit. Care Med..

[31] Leng L., Wang C., Deng Y., Hu Y. (2024). Identification of the Key Targets for Sepsis-Associated Acute Kidney Injury by RNA Sequencing Combined with Bioinformatics Methods. BMC Immunol..

[32] Klocke J., Kim S.J., Skopnik C.M., Hinze C., Boltengagen A., Metzke D., Grothgar E., Prskalo L., Wagner L., Freund P. (2022). Urinary Single-Cell Sequencing Captures Kidney Injury and Repair Processes in Human Acute Kidney Injury. Kidney Int..

[33] Han R., Li W., Tian H., Zhao Y., Zhang H., Pan W., Wang X., Xu L., Ma Z., Bao Z. (2023). Urinary microRNAs in Sepsis Function as a Novel Prognostic Marker. Exp. Ther. Med..

[34] Lysak N., Ebadi A., Bandyopadhyay S., Ozrazgat-Baslanti T., Sautina L., Lopez M.-C., Baker H.V., Segal M.S., Bihorac A. (2017). Unsupervised Machine Learning Analysis of Urinary Transcriptome Reveals Distinct Genotypic Clustering in Surgical Sepsis. J. Am. Coll. Surg..

[35] Bandyopadhyay S., Lysak N., Adhikari L., Velez L.M., Sautina L., Mohandas R., Lopez M.-C., Ungaro R., Peng Y.-C., Kadri F. (2020). Discovery and Validation of Urinary Molecular Signature of Early Sepsis. Crit. Care Explor..

[36] Bandyopadhyay S., Loftus T.J., Peng Y.-C., Lopez M.-C., Baker H.V., Segal M.S., Graim K., Ozrazgat-Baslanti T., Rashidi P., Bihorac A. (2022). Early Differentiation Between Sepsis and Sterile Inflammation via Urinary Gene Signatures of Metabolic Dysregulation. Shock.

[37] Kiseļova T., Vilne B., Zvīgule-Neidere G., Gardovska D. (2025). Exploratory Pilot Study on Transcriptome Analysis in Blood and Urine for Identifying Potential miRNA Biomarker Candidates to Dustinguish of Bacterial vs. Viral Infections in Children with Fever. medRxiv.

[38] R Core Team (2026). R: A Language and Environment for Statistical Computing.

[39] Love M.I., Huber W., Anders S. (2014). Moderated Estimation of Fold Change and Dispersion for RNA-Seq Data with DESeq2. Genome Biol..

[40] Benjamini Y., Hochberg Y. (1995). Controlling the False Discovery Rate: A Practical and Powerful Approach to Multiple Testing. J. R. Stat. Soc. Ser. B Stat. Methodol..

[41] Wu T., Hu E., Xu S., Chen M., Guo P., Dai Z., Feng T., Zhou L., Tang W., Zhan L. (2021). clusterProfiler 4.0: A Universal Enrichment Tool for Interpreting Omics Data. Innovation.

[42] Korotkevich G., Sukhov V., Budin N., Shpak B., Artyomov M.N., Sergushichev A. (2016). Fast Gene Set Enrichment Analysis. bioRxiv.

[43] Aleksander S.A., Balhoff J.P., Carbon S., Cherry J.M., Ebert D., Feuermann M., Gaudet P., Harris N.L., Hill D.P., The Gene Ontology Consortium (2026). The Gene Ontology Knowledgebase in 2026. Nucleic Acids Res..

[44] Kanehisa M., Furumichi M., Sato Y., Matsuura Y., Ishiguro-Watanabe M. (2025). KEGG: Biological Systems Database as a Model of the Real World. Nucleic Acids Res..

[45] Martens M., Ammar A., Riutta A., Waagmeester A., Slenter D.N., Hanspers K., Miller R.A., Digles D., Lopes E.N., Ehrhart F. (2021). WikiPathways: Connecting Communities. Nucleic Acids Res..

[46] Liberzon A., Birger C., Thorvaldsdóttir H., Ghandi M., Mesirov J.P., Tamayo P. (2015). The Molecular Signatures Database Hallmark Gene Set Collection. Cell Syst..

[47] Wickham H. (2016). Ggplot2.

[48] Liu S., Duan C., Xie J., Zhang J., Luo X., Wang Q., Liang X., Zhao X., Zhuang R., Zhao W. (2023). Peripheral Immune Cell Death in Sepsis Based on Bulk RNA and Single-Cell RNA Sequencing. Heliyon.

[49] Giannini H.M., Kan M., Cosgriff C.V., Morley M.P., Miano T.A., Narayanan N., Ittner C.A.G., Turner A.P., Esperanza M.P., Erlich M.C. (2026). Whole Blood Transcriptomics Reveals Sepsis Mortality-Associated Changes in Neutrophil Degranulation. Am. J. Respir. Cell Mol. Biol..

[50] Yang Y., Hua Y., Yang S., Liu N., Li J. (2026). Single-Cell Transcriptomic Analysis Deciphers Heterogeneity and Transcriptional Regulatory Programs of Sepsis with Different Prognosis. Biol. Direct.

[51] Chen I.-C., Chen H.-H., Jiang Y.-H., Hsiao T.-H., Ko T.-M., Chao W.-C. (2023). Whole Transcriptome Analysis to Explore the Impaired Immunological Features in Critically Ill Elderly Patients with Sepsis. J. Transl. Med..

[52] Cheng P.-L., Chen H.-H., Jiang Y.-H., Hsiao T.-H., Wang C.-Y., Wu C.-L., Ko T.-M., Chao W.-C. (2021). Using RNA-Seq to Investigate Immune-Metabolism Features in Immunocompromised Patients With Sepsis. Front. Med..

[53] Darden D.B., Dong X., Brusko M.A., Kelly L., Fenner B., Rincon J.C., Dirain M.L., Ungaro R., Nacionales D.C., Gauthier M. (2021). A Novel Single Cell RNA-Seq Analysis of Non-Myeloid Circulating Cells in Late Sepsis. Front. Immunol..

[54] Washburn M.L., Wang Z., Walton A.H., Goedegebuure S.P., Figueroa D.J., Van Horn S., Grossman J., Remlinger K., Madsen H., Brown J. (2019). T Cell– and Monocyte-Specific RNA-Sequencing Analysis in Septic and Nonseptic Critically Ill Patients and in Patients with Cancer. J. Immunol..

[55] Zhang Q., Hu Y., Wei P., Shi L., Shi L., Li J., Zhao Y., Chen Y., Zhang X., Ye F. (2022). Identification of Hub Genes for Adult Patients with Sepsis via RNA Sequencing. Sci. Rep..

[56] Reyes M., Filbin M.R., Bhattacharyya R.P., Billman K., Eisenhaure T., Hung D.T., Levy B.D., Baron R.M., Blainey P.C., Goldberg M.B. (2020). An Immune-Cell Signature of Bacterial Sepsis. Nat. Med..

[57] Chenoweth J.G., Colantuoni C., Striegel D.A., Genzor P., Brandsma J., Blair P.W., Krishnan S., Chiyka E., Fazli M., Mehta R. (2024). Gene Expression Signatures in Blood from a West African Sepsis Cohort Define Host Response Phenotypes. Nat. Commun..

[58] Middleton E.A., Rowley J.W., Campbell R.A., Grissom C.K., Brown S.M., Beesley S.J., Schwertz H., Kosaka Y., Manne B.K., Krauel K. (2019). Sepsis Alters the Transcriptional and Translational Landscape of Human and Murine Platelets. Blood.

[59] Li N., Tang T.-T., Gu M., Fu Y.-Q., Qian W.-W., Ma N.N., Shen A.-R., Zhu T., Huo R.-B., Zhang T. (2025). Single Urinary Extracellular Vesicle Proteomics Identifies Complement Receptor CD35 as a Biomarker for Sepsis-Associated Acute Kidney Injury. Nat. Commun..

[60] Luxen M., Zwiers P.J., Jongman R.M., Moser J., Pultar M., Skalicky S., Diendorfer A.B., Hackl M., Van Meurs M., Molema G. (2024). Sepsis Induces Heterogeneous Transcription of Coagulation- and Inflammation-Associated Genes in Renal Microvasculature. Thromb. Res..

[61] Yuan N., Chen J., Luo F., Fang Z., Huang M., Liu J., Chen X., Luo C. (2025). Comparison of Sepsis-Associated Acute Kidney Injury with Different Degrees and Causes Reveals Patterns in Mitochondrial Metabolism and Immune Infiltration Changes. Sci. Rep..

[62] Janosevic D., Myslinski J., McCarthy T.W., Zollman A., Syed F., Xuei X., Gao H., Liu Y.-L., Collins K.S., Cheng Y.-H. (2021). The Orchestrated Cellular and Molecular Responses of the Kidney to Endotoxin Define a Precise Sepsis Timeline. eLife.

[63] Zhang Z., Chen L., Liu H., Sun Y., Shui P., Gao J., Wang D., Jiang H., Li Y., Chen K. (2022). Gene Signature for the Prediction of the Trajectories of Sepsis-Induced Acute Kidney Injury. Crit. Care.

[64] Zhang Z., Xu F. (2026). Immunotherapy for Sepsis: From Single-Cell Scenarios to Clinical Translation. Genes Dis..

